# Salivary gland cancers in elderly patients: challenges and therapeutic strategies

**DOI:** 10.3389/fonc.2022.1032471

**Published:** 2022-11-25

**Authors:** Elena Colombo, Charlotte Van Lierde, Alexandra Zlate, Alexandra Jensen, Gemma Gatta, Fabio Didonè, Lisa F. Licitra, Vincent Grégoire, Vander Vander Poorten, Laura D. Locati

**Affiliations:** ^1^ Head and Neck Medical Oncology Unit, Fondazione IRCCS Istituto Nazionale dei Tumori, Milan, Italy; ^2^ Otorhinolaryngology-Head and Neck Surgery, Leuven Cancer Institute, University Hospitals Leuven and Department of Oncology, section Head and Neck Oncology, KU Leuven, Leuven, Belgium; ^3^ Department of Radiation Oncology, Centre Leon Berard, Lyon, France; ^4^ Department of Radiation Oncology, University Hospitals Giessen and Marburg (UKGM), Marburg, Germany; ^5^ Evaluative Epidemiology Unit, Fondazione IRCCS Istituto Nazionale dei Tumori, Milan, Italy; ^6^ Department of Oncology and Hematology, University of Milan, Milan, Italy

**Keywords:** salivary gland (SG) tumors, geriatric oncology, salivary gland surgery, radiation therapy (radiotherapy), particle therapy, precision oncology, multidisciplinary team (MDT)

## Abstract

Salivary gland carcinomas (SGCs) are the most heterogeneous subgroup of head and neck malignant tumors, accounting for more than 20 subtypes. The median age of SGC diagnosis is expected to rise in the following decades, leading to crucial clinical challenges in geriatric oncology. Elderly patients, in comparison with patients aged below 65 years, are generally considered less amenable to receiving state-of-the-art curative treatments for localized disease, such as surgery and radiation/particle therapy. In the advanced setting, chemotherapy regimens are often dampened by the consideration of cardiovascular and renal comorbidities. Nevertheless, the elderly population encompasses a broad spectrum of functionalities. In the last decades, some screening tools (e.g. the G8 questionnaire) have been developed to identify those subjects who should receive a multidimensional geriatric assessment, to answer the question about the feasibility of complex treatments. In the present article, we discuss the most frequent SGC histologies diagnosed in the elderly population and the relative 5-years survival outcomes based on the most recent data from the Surveillance, Epidemiology, and End Results (SEER) Program. Moreover, we review the therapeutic strategies currently available for locoregionally advanced and metastatic disease, taking into account the recent advances in precision oncology. The synergy between the Multidisciplinary Tumor Board and the Geriatrician aims to shape the most appropriate treatment pathway for each elderly patient, focusing on global functionality instead of the sole chronological age.

## 1 Introduction

The global population is ageing at a fast pace. In 2050, the proportion of the world’s population over 60 years is expected to reach 22%, almost doubling the 12% of 2015 (1). The World Health Organization (WHO) defines *elderly* the subjects aged beyond 65 years (65y+), a highly heterogeneous group of people in terms of performance status, comorbidities and vulnerabilities.

The variability of elderly patients poses significant challenges to the clinical practice of oncology (2, 3), including the context of rare cancers, where few clinical trials are available and study populations are less numerous (4). Clinicians have few tools to estimate the risk/benefit balance of treatments for elderly patients, since an age subgroup-specific analysis is unlikely feasible. However, the differences in survival outcomes from rare cancers in the United States and Europe are more evident for age group rather than for cancer type, with the 65y+ subjects at high risk of poor outcome (5).

The level of clinical challenge is major for patients with rare tumors requiring a multidisciplinary approach, as in the case of head and neck cancers (HNCs). In patients with HNCs, both age and comorbidities influence the overall survival (OS), possibly because of advanced tumor stage, inability to perform multimodal treatments and/or non cancer-related causes of death (6). Moreover, the consideration of chronological age and mild/moderate comorbidities may result in the selection of substandard treatments, which are associated to lower OS and cancer-specific survival, in comparison with the state-of-the-art (7). However, HNCs encompass a heterogeneous group of cancers with different histologies and clinical courses. This review is focused on salivary gland cancers (SGCs) in elderly patients, including the most recent epidemiological findings, the current treatment approaches and the future perspectives.

SGCs are rare cancers, accounting for less than 5% of all malignancies of the cervicofacial region. The WHO Global Cancer Observatory (Globocan) reported 53.583 new cases of SGCs diagnosed in 2020 worldwide, 43% occurring in the elderly, and causing 12.339 cancer-specific deaths, with a male-to-female ratio of 1.3:1. In the next two decades, the new diagnoses in the elderly age group are expected to account for 80% of the total SGCs diagnoses; similarly, SGCs in this group are expected to cause the 88% of all SGC-specific deaths (8). The impact of SGCs in the elderly is going to become a crucial health issue, thus research efforts should be encouraged to identify the main risk factors and the most effective therapeutic strategies for this multifaceted population.

The ICARE study, a multicenter, population-based, case-control study recently conducted in France on 73 SCGs and 3555 controls, reported that the main risk factors for SGCs were related to a previous history of cervicofacial radiation therapy (RT), either for HNCs (odds ratio, OR = 31.74, 95% CI 2.5 – 405.2) or hematological cancers (OR = 5.1, 95% CI 0.6 – 46.2), and professional exposure to chemicals, such as metals in the plumbing industry, electrical equipment and nickel compounds/alloy. In the ICARE study, the mean age at diagnosis was 56.9 years, with 27% of the population aged more than 62 years (9). Another case-control study conducted in Japan showed that heavy smokers were at higher risk of developing SGCs, compared with never smokers (OR = 3.45, 95% CI 2.06-12.87; p < 0.001); 43.7% of the study population was more than 60 years of age (10).

On the basis of the current literature, it is unknown whether the onset of SGCs in the elderly population could be sustained by risk factors that are different from those of younger patients. However, some differences can be observed not only in the survival outcomes, but also in the histotype-specific incidence. The clinical management of elderly patients has improved in the last decades, due to new clinical tools of geriatric assessment. The Comprehensive Geriatric Assessment (CGA) is a method for identifying elderly patients at risk of life-threatening events during oncological therapy by analyzing several domains (functionality, nutrition, cognition, psychological state, social support, comorbidities, medication review, and geriatric syndromes). It can predict functional decline and also be used to adapt cancer treatment, as demonstrated by the ELCAPA study (11). Since CGA is a time-consuming tool, in the last decade various screening instruments were adopted, in order to refine the selection of vulnerable patients who could benefit from a CGA. The G8 questionnaire, based on seven items from the Mini Nutritional Assessment (MNA) and age as the 8^th^ item, uses a scoring system from 0 to 17, where a result below the cut-off value of 14 identifies those patients who should be addressed to CGA. G8 has been validated in multicenter cohort studies (12, 13) and also in a population of elderly HNSCC patients treated with chemo(radio)therapy (14). G8 has high sensitivity, but a proportion of patients with G8 scores ≤14 has no major vulnerabilities detected by the CGA. Moreover, the level of G8 specificity may vary according to the primary tumor sites, as demonstrated by the ELCAPA-02 study, which included few patients with HNC (n=4) (15). An optimized version of the G8 was recently proposed, including six independent predictors for abnormal CGA: weight loss, cognition/mood, performance status, self-rated health status, polypharmacy (≥ 6 medications per day), and history of heart failure/coronary heart disease (16).

Focusing on the treatment of patients with SGCs, the recently released ASCO Guidelines recommend taking clinical decisions in the context of a multidisciplinary tumor board, focusing on histology, disease burden and site of tumor deposits, potential treatment-related toxicities, patient’s overall health, comorbidities and function (17). Comorbidities are the Achilles’ heel of elderly patients with cancer, however, patients with SGCs have less comorbidities compared to those with other HNCs. In a retrospectively analyzed cohort of 666 patients with SGCs, OS – but not disease-free survival (DFS) – was influenced by the Adult Comorbidity Evaluation index (ACE-27). The ACE-27 scoring was affected by age and gender, possibly influenced by lifestyle. In that study, patients with comorbidities were more likely to receive a non-surgical treatment, i.e. exclusive RT, or no treatment at all, achieving a worse outcome. The impact of comorbidities was not related to the histological subtypes, except for the squamous cell histology. Interestingly, DFS was not influenced by comorbidities, indicating that a proper treatment should be delivered as much as possible, even in elderly patients with comorbidities (18).

Another study focused on the influence of age and comorbidities on the outcome using the Age-Adjusted Charlson Comorbidity Index (ACCI) scoring system on a series of 109 patients with a median age of 69 years, treated for major SGCs. Comorbidities, but not age, were an independent prognostic factor for both OS and disease-specific survival (19). A Danish study performed in 871 patients with SGC treated between 1990 and 2005, reported a poorer survival in the population group over 70 years old (n=282), possibly explained by the more advanced disease stages, poorer performance status at the time of diagnosis, more high-grade histological subtypes such as adenocarcinoma not otherwise specified (NOS) and carcinoma ex-pleomorphic adenoma (ca ex-PA). Despite these aggressive clinical features, elderly patients received surgery plus RT only in 45% of cases, while 54% received a suboptimal active treatment, either surgery alone (38%) or exclusive RT (10%), or best supportive care (6%) (20).

Malignant tumors of the major salivary glands (MSG) can be found in 15-32% of parotid, 41-45% of submandibular and 70-90% of sublingual masses. The minor salivary glands (mSG) are located beneath the mucosa of the oral cavity, palate, paranasal sinuses, pharynx, larynx, trachea and bronchi, mostly concentrated in the buccal, labial, palatal, and lingual regions. Almost 50% of the tumors arising from mSG are malignant (21). The staging of MSG is currently based on the 8^th^ edition of the American Joint Committee on Cancer/Union for International Cancer Control (AJCC/UICC) tumor/node/metastasis (TNM) System, while mSGCs are staged according to the AJCC/UICC system for the primary site (22). In patients with a suspicion of SGC, the ASCO Guidelines recommend performing an imaging workup including neck ultrasound, computed tomography (CT) with intravenous contrast, and/or magnetic resonance imaging (MRI) of the neck and primary site (Recommendation 1.1) (17). An MRI with diffusion sequence of the neck and skull base is recommended in case of suspicious perineural spread and/or skull base involvement (R1.3), and an MRI brain scan could be considered in case of high-grade SGCs or if suspected meningeal spread. A CT scan should be performed in case of possible involvement of the adjacent bone (R1.2). An FDG-PET/CT scan from the skull base to mid-thighs may be performed for tumors with high-grade features, while in low-grade histological subtypes it could deliver false negative results (R1.4). For locoregionally advanced cases, a contrast-enhanced chest-abdomen CT scan is recommended to complete the clinical staging. Intracranial metastases are infrequent at diagnosis; however, they may occur especially in case of high-grade SGC (23).

**Figure 1:**
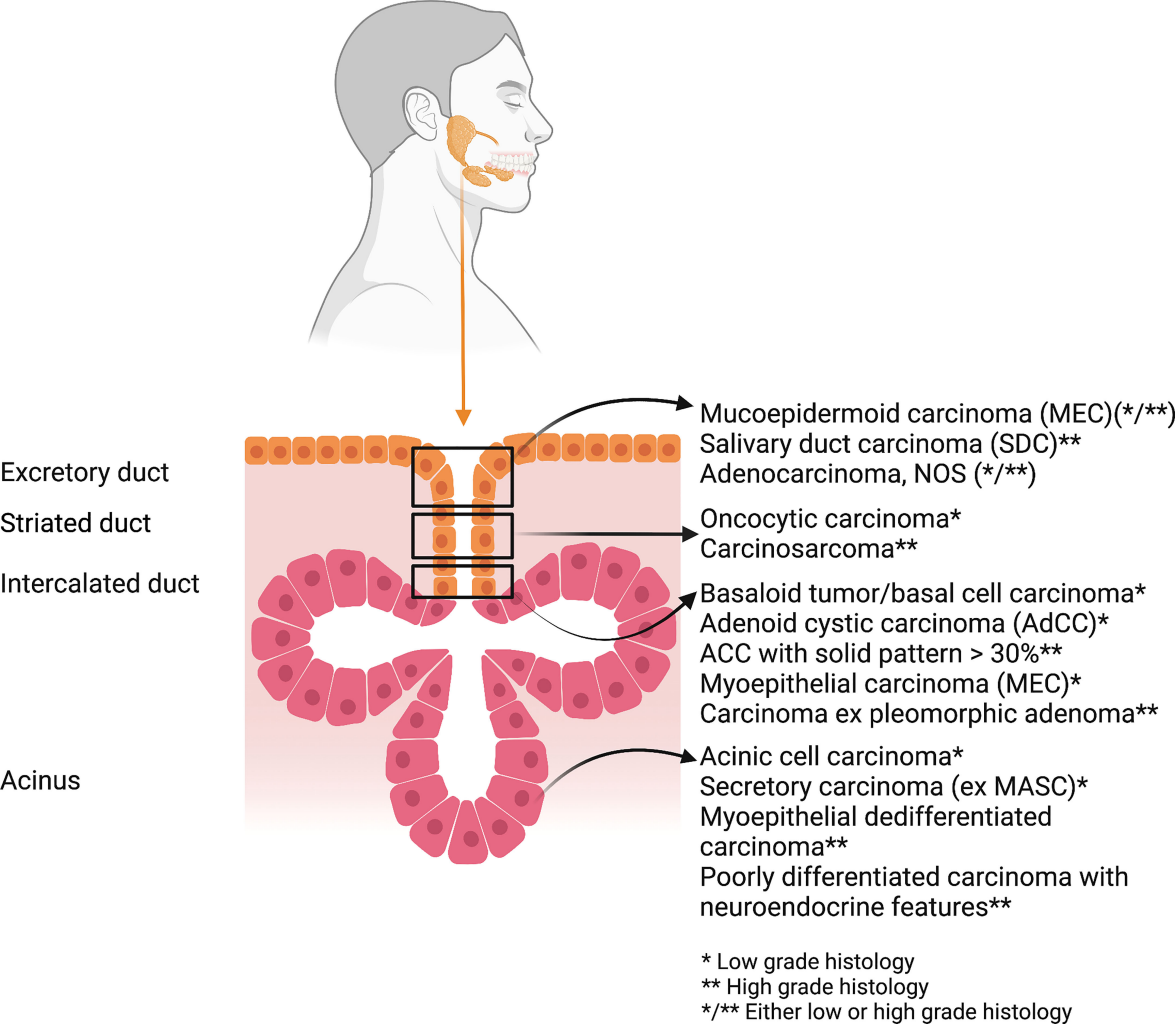
The most frequent histotypes of salivary gland cancers stratified according to the glandular portion of origin and the prevalent grading (*low grade; ** high grade; */** either low- or high-grade histology). Graphic created with BioRender.com.

Beside the multiplicity of anatomical subsites, SGCs harbor a wide heterogeneity of histologies, which are associated to different age incidence, clinical behavior, treatments and prognosis. Each glandular segment can be the site of origin of SGCs (**Figure 1**). The WHO 2017 Classification of Salivary Gland Tumors (4^th^ Edition) classified more than 20 malignant subtypes of epithelial SGCs, including a new entity, the secretory carcinoma (SC), formerly known as mammary analogue SC (MASC) (24, 25). Moreover, the recently released Armed Forces Institute of Pathology (AFIP) Atlas of SGC Tumor Pathology provides an indepth analysis of the morphological and molecular features of both benign and malignant salivary gland tumors (26).

Recently, the molecular landscape of SGCs has been explored by comprehensive genomic profiling, opening this complex disease to the possibilities of targeted therapy (27, 28). Molecular alterations found in SGCs include amplifications, mutations or rearrangements of transmembrane receptors (*ERBB2, FGFR, PDGFR, RET*), mTOR pathway (*PIK3CA, PTEN*) and MAPK pathway (*BRAF, HRAS*), DNA repair (*BRCA1/2*), cycle cell regulation (*CDKN2A/B, SMARCB1*) and activation of androgen-responsive genes by the androgen receptor (AR). Interestingly, the upregulation of *ERBB2*/*PIK3CA* pathways is more frequently observed in high-grade than in low-grade SGCs (27). However, certain histotypes can encompass both low-grade and high-grade forms, as in the case of polymorphous adenocarcinoma (PAC), previously known as polymorphous low-grade adenocarcinoma (PLGA); in the last 2017 WHO classification of Head and Neck tumors, both the classic variant of PLGA and aggressive cribriform adenocarcinoma of minor salivary glands (CAMSG), were incorporated under the PAC category. Interestingly, both the variants harbor in the majority of cases an alteration of *PRKD* genes codifying for Serine/Threonine-Protein Kinase D1, most frequently mutations in the classic PAC variant and rearrangements in CAMSG (29). Certain key rearrangements, such as *MYB-NFIB*, *NR4A3*, *PLAG1*, *ETV6-NTRK/RET*, *CRTC1-MAML2* are typical of certain histotypes, and may help pathologists in the diagnosis of challenging cases (30–36) (**Table 1**).

**Table 1:** Common molecular alterations of the most frequent types of SGCs.

SGC Histotype	Grade	Mutations[% cases]	Gene fusions/amplifications	Chromosomal alterations	Ref.
Myoepithelial carcinoma (MyoEpi)	Low	***CDKN2A* [25]** ***HRAS* [20-25]** ***CDKN2B* [25]** ***PIK3CA* [15]** ***RICTOR* [15]** ***NOTCH1* [15]** ***PTCH1* [10]** ***PDGFRB* [5]** ***FGFR2* [5]**	***PLAG1* [26]** • *LIFR-PLAG1* • *CTNNB1-PLAG1*		(27, 31)
Secretory carcinoma (SC), formerly mammary analogue SC (MASC)		–	***ETV6-NTRK*** ***ETV6-RET*** ***VIM-RET*** ***ETV6-MET***	t(12; 15) t(12; X)	(33–36)
Acinic cell carcinoma (AcCC)	Low/ High (rare)	***CDKN2A* [75]** ***CDKN2B* [45]** ***PTEN* [10]** ***HRAS* [5]** ***BRAF* [5]**	***NR4A3*** upregulation	t(4; 9)	(27, 30, 37)
Adenoid cystic carcinoma (AdCC)	Low/ High (rare)	***NOTCH* [25]** ***PIK3CA* [5]** ***PDGFRA* [5]** ***CDKN2A* [5]** ***RET* [<5]** ***FGFR2* [<5]**	*MYB-NFIB* [60-80] *MYBL1-NFIB*	t(6; 9) t(8; 9)	(27, 30, 38)
Polymorphous adenocarcinoma (PAC)	Low/ High (rare)	***PRKD1* [80]** ***FGFR1* [20]** ***PTEN* [20]** ***TSC2* [20]**	*PRKD1/2/3* • *ARID1A-PRKD1* • *DDX3X-PRKD1*		(27)
Mucoepidermoid carcinoma (MEC)	High	***CDKN2A* [45]** ***PIK3CA* [20]** ***HRAS* [10]** ***BRCA1*/2 [10]** ***FGFR1* [5-10]** ***PTEN* [5-10]** ***BRAF* [5]**	***CRTC1-MAML2*** ***CRTC3-MAML2*** ***EWSR1-POU5F1*** ***ERBB2*** ampl. [10]	t(11; 15) t(11; 19) t(6; 22) 17q21.1	(27, 30)
Salivary duct carcinoma (SDC)		***PIK3CA* [25]** ***HRAS* [20]** ***CDKN2A* [15]** ***PTEN* [15]** ***BRAF* [5-10]** ***BRCA1*/2 [5]**	***ERBB2*** ampl. [30] ***AR*** ampl.	17q21.1	(27)
Adenocarcinoma, NOS (AD-NOS)		***PIK3CA* [25]** ***HRAS* [20]** ***CDKN2A* [15]** ***PTEN* [10-15]** ***RET* [5-10]** ***BRAF* [5-10]**	***ERBB2*** ampl. [15] *AR* ampl.	17q21.1	(27)
Carcinoma ex pleomorphic adenoma (ca ex-PA)		***PTEN* [10-15]** ***FGFR1* [10]** ***FGFR2* [10]** ***SMARCB1* [10]** ***NOTCH1* [10]** ***BRCA1* [5]**	***ERBB2*** ampl. [30] ***PLAG1*** • *FGFR1-PLAG1* • *CTNNB1-PLAG1* • *CHCHD7-PLAG1*	17q21.1	(27, 32)

The most frequent high-grade histotypes are mucoepidermoid carcinoma (MEC), salivary duct carcinoma (SDC), adenocarcinoma NOS (AD-NOS) and carcinoma ex-pleomorphic adenoma (ca ex-PA). Low-grade histotypes include adenoid cystic carcinoma (AdCC), acinic cell carcinoma (AcCC), myoepithelial carcinoma (MyoEpi), and SC (previously MASC). However, both AdCC and AcCC may develop a high-grade transformation by dedifferentiation, and this phenomenon has been described also in elderly patients (37, 38) (**Table 1**).

Tumor grading is directly related to the disease aggressiveness, and it is of utmost interest to capture the age distribution of the commonest types of SGCs. As reported in the AFIP Atlas, certain histotypes are more frequently diagnosed in the 6^th^ decade and beyond, especially SDC, AcCC with high-grade transformation, MyoEpi, basal cell adenocarcinoma, primary squamous cell carcinoma (SCC), large cell undifferentiated carcinoma, epithelial-myoepithelial carcinoma, and carcinosarcoma (26).

## 2 Epidemiology

For the purpose of this review, we report the age-stratified incidence of epithelial SGCs using the Surveillance, Epidemiology, and End Results (SEER) Program database provided by the U.S.A. National Cancer Institute’s Division of Cancer Control and Population Sciences (39). We selected the cases diagnosed between 2011 and 2018 and retrieved from the updated dataset of the SEER Program based on 18 registries from 13 States (88.816.582 inhabitants). SGCs cases were defined using a combination of the International Classification of Diseases for Oncology (ICD-O) morphology and topography codes (40), as proposed by the RARECAREnet project (5). The SGCs included in the SEER database were divided into two groups:

1 - malignant epithelial tumors of the major salivary glands (MSG);

2 - malignant epithelial salivary glands tumors other than MSG (SGT).

In both groups we report the incidence and 5-year survival (%) by morphology codes, stage at diagnosis and age groups (0-65y and 65y+) (**Tables 2**–**4**). The methods used for the calculation of the frequency (incidence) and outcome (relative survival) were provided by the SEERStat program. The number of new diagnoses made in one year for each specific population of cases (incidence) was expressed in annual rate, defined as the number of cases on 100,000 inhabitants per year. The relative survival is the ratio between life expectancy of the cohort of cases affected by cancer and the life expectancy of the population of cases. It is the closest estimation of cause-specific survival in clinical studies.

**Table 2:** Incidence rates of SGCs per 100.000 people, frequency and number by age and histotype. a) Major salivary glands tumors (MSG); b) HN salivary glands tumors (SGT) other than MSG. Period of diagnosis 2011-2018.

Histotype	0–65y				65y+				Total	
a – Major Salivary Gland (MSG) Tumors	Count	Rate		%	Count		Rate	%	Count	Rate
Neoplasms NOS*	257	0,042		6%	605		0,623	12%	862	0,121
SCC	456	0,074		10%	1645		1,695	32%	2101	0,296
AD-NOS	466	0,076		10%	669		0,689	13%	1135	0,160
AdCC	520	0,085		11%	371		0,382	7%	891	0,125
MEC	1355	0,221		30%	738		0,760	14%	2093	0,295
SDC	253	0,041		6%	234		0,241	5%	487	0,069
AcCC	726	0,118		16%	288		0,297	6%	1014	0,143
Epi-MyoEpi	94	0,015		2%	130		0,134	3%	224	0,032
Ca ex-PA	175	0,029		4%	176		0,181	3%	351	0,049
Malignant myoepithelioma	75	0,012		2%	90		0,093	2%	165	0,023
Other histotypes	187	0,030		4%	212		0,218	4%	399	0,056
**Total**	4564	0,744		100%	5158		5,315	100%	9722	1,368
b – Salivary Gland Tumors (SGT) excluding MSG										
AD-NOS	236	0,038		12%	257		0,265	21%	493	0,069
AdCC	553	0,090		29%	362		0,373	29%	915	0,129
Clear cell adenocarcinoma, NOS	48	0,008		2%	24		0,025	2%	72	0,010
MEC	727	0,119		38%	328		0,338	27%	1055	0,148
PAC	193	0,031		10%	140		0,144	11%	333	0,047
AcCC	40	0,007		2%	14		0,014	1%	54	0,008
Other histotypes	125	0,020		7%	105		0,108	9%	230	0,032
**Total**	1922	0,313		100%	1230		1,267	100%	3152	0,444

*NOS, not otherwise specified (ICD morphology codes 8000-8010).

**Table 3:** Five-years relative survival rates (5y-RS) by age and histotype for a) major salivary glands tumors (MSG), b) Salivary glands type tumors (SGT) other than MSG. .

Histotypes	All ages	0–65y	65y+	Delta
a – MSG			
Neoplasms NOS*	43%	60%	34%	-26%
SCC	44%	53%	45%	-8%
AD-NOS	64%	71%	57%	-14%
BCA	98%	100%	93%	-7%
AdCC	77%	80%	72%	-8%
MEC	89%	93%	78%	-15%
SDC	64%	60%	68%	8%
SC (ex MASC)	97%	96%	96%	0%
AcCC	93%	94%	89%	-5%
Epi-MyoEpi	97%	95%	97%	2%
Ca ex-PA	83%	79%	86%	7%
Malignant myoepithelioma	85%	83%	86%	3%
**Total**	72%	82%	61%	-21%
**b - SGT**				
AD-NOS	71%	76%	64%	-12%
AdCC	78%	81%	73%	-8%
Clear cell adenocarcinoma, NOS	90%	83%	100%	17%
MEC	93%	95%	84%	-11%
PAC	99%	98%	99%	1%
AcCC	94%	97%	81%	-16%
**Total**	86%	89%	81%	-8%

Years of diagnosis: 2011-2018. The histotypes with the broadest survival delta between elderly and younger populations are highlighted in red color.

*neoplasms not otherwise specified =ICD morphology codes 8000-8010.

**Table 4:** Five-years relative survival (5y-RS) after MSG and SGT diagnoses, stratified by age (0–65y vs 65y+) and extent of disease (localized, locoregional, metastatic, unknown) expressed in raw numbers and in percentage on the total number of cases.

Extent of Disease	All ages			0–65y			65y+			Delta5y-RS
MSG	N	cases (%)	5y-RS (%)	N	cases (%)	5y-RS (%)	N	cases (%)	5y-RS (%)	
Localized	3408	48	93.9	2123	57	96.8	1285	38	88.5	-8.3
Locoregional	2051	29	65.0	918	24	**75.9**	1133	34	**54.7**	**-21.2**
Metastatic	1291	18	35.4	572	15	**42.5**	719	21	**28.8**	**-13.7**
Unknown	381	5	55.0	143	4	**70.0**	238	7	**44.2**	**-25.8**
**Total cases**	7131	100	72.4	3756	100	81.5	3375	100	61.3	-20.2
**SGT**	
Localized	1346	52	99.2	902	53	99.7	444	50	97.9	-1.8
Locoregional	622	24	79.0	400	24	82.2	222	25	73.0	-9.2
Metastatic	428	17	53.6	280	17	**63.2**	148	17	**34.3**	**-28.9**
Unknown	174	7	81.8	107	6	**90.6**	67	8	**64.6**	**-26.0**
**Total cases**	1346	52	99.2	902	53	99.7	444	50	97.9	-1.8

In comparison with 0-65y subgroup, in the 65y+ the incidence rates were 4 and 7 times higher for SGT and MSG, respectively. Focusing on the histotypes, AcCC, MEC and AdCC of SGT were more frequently diagnosed in the 0-64y cohort, while SCC were more common in elderly patients with MSG. Moreover, the neoplasms NOS of MSG and AD-NOS of SGT were more commonly diagnosed in the elderly, compared with younger patients (70% vs 30%) (**Table 2**).

In the SEER dataset population, the overall 5-years survival rates were significantly better in young than in elderly subjects: the survival rates after MSG and SGT diagnoses were 82% and 89% in the 0-65y group, 61% and 81% in the 65y+ group. Interestingly, the worst outcomes were reported for elderly patients with MSG. The 5-years survival rate was lower for elderly patients in the majority of histotypes, especially in neoplasms NOS (34%), AD-NOS (57%), MEC (78%) of the MSGs and AcCC (81%) of the SGT (**Table 3**). The differences in survival outcomes observed between the two age groups may be partially explained by a higher proportion of histotypes with favorable prognosis in the 0-65y group, compared to the 65y+ (**Table 2**).

Almost half of SGC cases from the SEER dataset presented at diagnosis with localised disease, without significant differences between MSG and SGT (**Table 4**). Elderly patients were diagnosed more frequently at a metastatic stage, and the 5-year survival for each stage was lower in the elderly subgroup. Focusing on the cases with available staging, the greatest difference between age subgroups could be observed for locoregional disease in MSG, where the multidisciplinary team is crucial to define an optimal management.

## 3 Surgery

### 3.1 State-of-the-art of surgical treatment

There is broad consensus that patients with resectable SGCs need surgery as an essential keystone to achieve cure. Depending on the postoperative pathological findings, the majority of patients need postoperative RT to maximize the chance of cure (17, 41–44). Regardless of age, the patients unfit for surgery and those where surgery is not expected to remove all the macroscopic disease are candidate to primary curative or palliative RT.

Independent prognostic factors for survival outcomes following locoregional SGC treatments have been well studied and validated in previous works (45, 46). Independently of the treatment modality, poor outcomes are seen with increasing TNM Stage (reflecting anatomical tumor extension and facial nerve function), higher histological grade, and surgical margins involvement. Increasing age is associated with more advanced stage tumors and more aggressive/high-grade histologies, where free surgical margins are more difficult to obtain. These features occur more frequently in elderly patients, and both biological age and comorbidities are negative prognostic factors (18, 21, 41, 47). Therefore, the most challenging SGCs occur in elderly patients (especially 70y+), who should receive a resection with free margins (R0), but often surgeons and the MDT may hesitate to recommend this important part of the curative treatment because of advanced age, decreased coping mechanisms and comorbidities.

### 3.2 Surgical approach in the elderly patient: biological age versus chronological age

The surgical anatomy of SGs is complex and resection can seriously interfere with vital functions such as speech and swallowing, leading to potentially major functional impact. Thus, it is important to tailor the treatment to the patient (17). Elderly patients, even when apparently still functioning well, are marked by a lower reserve of physiological resources to cope with a surgical treatment and its expected consequences. Frailty is a continuous variable; it reflects the general physical and mental conditions and is directly related to the “biological age” (48–50). For patients affected by HNC, chronological age is not an absolute contraindication for surgery, as it is not strictly associated with major complications. Nevertheless, comorbidities are important predictors of outcome and should be taken into account by the MDT (51). The G8 questionnaire is a valid screening tool to identify elderly patients who need a comprehensive geriatric assessment, leading to an intensified perioperative care (48–50, 52). An impaired G8 score (≤ 14) predicts a prolonged hospital stay, higher risk of delirium and 1-year mortality (53).

Usually, patients with SGC do not present the same risk factors as the population with HNSCC, such as smoke habit and/or alcohol consumption; at equal chronological age, they have lower biological age, they are less frail and have lower ACE-27 scores (18, 48). This translates in a limited role of chronological age in the decision-making process for the elderly patient with SGC, in comparison with the typical HNSCC patient, and less restraint to offer surgery. The feasibility of this approach was proven by a large Dutch cohort where OS, as expected, depended on the ACE-27 score, but DFS did not, meaning that elderly patients with comorbidities received largely the same treatment as the other patients, and had a comparable chance for cure (18). Furthermore, a recent study on the effect of the Age Adjusted Charlson Comorbidity Index (ACCI), incorporating chronological age in the Comorbidity score), in patients with SGCs found that comorbidity did not influence the extent of cancer therapy, and 97% of patients had surgery as initial treatment in this cohort. Patients with a high ACCI score (> 4) in this study had worse oncological and survival outcomes (19).

The following paragraphs provide subsite-dependent state-of-the-art surgical considerations and potential comorbidity-related nuances for operable elderly patients, bearing in mind the limited amount of studies specifically dedicated to elderly patients.

### 3.3 Surgical resection of the primary tumor according to anatomical subsites

#### 3.3.1 Parotid glands

For parotid SGC, a nerve preserving parotidectomy should be performed whenever the facial nerve is preoperatively functioning and detachable from the tumor. If unavoidable, microscopic tumor remnants left on a functioning nerve can be controlled by adjuvant RT. These principles apply also to elderly patients (21, 41, 43). Regarding the extent of parotidectomy, for high-grade T1-T2 tumors localized in the superficial parotid lobe there is still no consensus on the opportunity of removing also the deep lobe (41). Some argue that being more conservative for small high-grade SGC, and removing only the involved part of the superficial lobe, reduces the risk of facial nerve damage without compromising the oncologic outcome (54), while others support total parotidectomy to address the intraparotid lymph nodes both in superficial and deep lobes (55–58). In the absence of high-quality evidence, it is possible to defend a more conservative approach in the 70y+ population, especially if the ACCI score is high (>4). Unfortunately, early-stage high-grade tumors are rarely encountered in this age group: more frequently, elderly patients are diagnosed with advanced-stage high-grade SGC infiltrating the facial nerve or the surrounding structures. Therefore, relatively often, an extended radical parotidectomy with facial nerve, skin, skull base or temporal bone resection is needed (41). As stated previously, chronological age should not impede the surgeon from performing this type of radical intervention, but a G8 geriatric assessment is required to estimate, and eventually increase, the coping capacities of elderly patients. Focusing on the long-term outcomes, the age plays a significant role. A 5-years disease-free survival prognostic model for patients with resected parotid carcinoma was externally validated on a population composed for 35% by 70y+ patients, reporting a significant association between high-grade histology and advanced age, perineural growth, non-radical resection, T classification and N classification. Combined in a prognostic model, all these factors provided more information on the outcome than histology. Therefore, the histology variable was not included in this prognostic index (59).

#### 3.3.2 Submandibular glands

Patients with T1-T2N0M0 submandibular SGCs are generally treated with surgery alone. Postoperative RT is needed in case of advanced stage, high grade, or pathological risk factors such as perineural invasion and/or positive resection margins (17, 20, 41, 42). Neck dissection of levels I-II-III is the minimal resection for a malignant submandibular SGC without evidence of lymph node involvement at clinical staging (cN0) (41, 47, 60). This should be taken into account especially for elderly patients presenting with high-grade SGCs or AdCC histology, which harbors a clinically occult pN+ rate of 1 out 4 (61). When preoperative MRI scan shows a tumor infiltrating the surrounding structures such as lingual, hypoglossal or marginal mandibular facial nerves, and muscles (digastric, stylohyoid, mylohyoid, hyoglossus), the MDT may deem it not feasible to operate frail elderly patients with comorbidities, while RT can preserve the neural function by achieving locoregional control. Conversely, an extended resection of nerves and mouth floor is usually well tolerated by fit elderly patients (21, 41, 42).

#### 3.3.3 Sublingual glands

Sublingual SGCs are extremely rare (41). A conservative resection, including Wharton’s duct and the submandibular gland, may be sufficient for small sublingual SGCs confined to the mouth floor (62). For tumors > 2 cm, a more aggressive en-bloc pull-through resection is indicated, due to the high rate of AdCC histology. The resection may need extension to involve the lingual nerve, a marginal mandibulectomy, when the tumor involves the periosteum, or a segmental mandibulectomy, if bone invasion is present. A level I-II-III neck dissection is indicated in these cases (63, 64). Comorbidities and frailty burden are more important than chronological age to assess whether the elderly patient will tolerate this type of surgery.

#### 3.3.4 Minor salivary glands

The primary treatment for resectable mSGC is surgery, most frequently with postoperative RT (44). Often, mSGC are not resectable when diagnosed at an advanced stage, especially if they have nasosinusal/nasopharyngeal origin. The most frequent histotype is AdCC, with a tendency to locally spread along nerves, subperiostal and perichondral planes (41, 44). Especially in elderly and frail patients, RT can achieve a good locoregional control and it should be preferred to complex multidisciplinary surgical efforts that would have profound functional impact, still requiring postoperative RT in the majority of cases. Interestingly, in recent research, postoperative complications were associated with frailty, but RT-associated toxicity was not (52). Moreover, in the context of unresectable mSGCs, particle therapy has presented better locoregional control than photon therapy (65), and this will be extensively discussed further.

### 3.4 Surgery of the neck with nodal involvement

Locoregionally advanced disease and high-grade tumors are frequently found in elderly patients (41, 66) and correlate with a high risk of relapse (17, 41, 45). In operable cases, independently of age and salivary gland of origin, the clinical evidence of nodal involvement (cN+) requires an ipsilateral neck dissection of levels I-V. Conversely, the surgical management of cN0 scenario is still debatable (41). For elderly patients, if there is a preoperatively assessed high risk of occult neck disease, valid options are both:

1. elective neck dissection associated with the primary surgery, based on a superselective level II dissection with frozen section (43, 67, 68);
2. elective neck irradiation, especially in frail patients, if adjuvant RT for the primary tumor is foreseen and confirmed by the pathological features of the primary tumor (69, 70).

### 3.5 Reconstruction following ablative parotid surgery in the elderly

Since advanced stages and high-grade tumors are frequently observed in the elderly, extended resections are often needed in this population. The resulting defects require a reconstruction to minimize the functional consequences and facilitate adjuvant RT (71). The reconstructing options for major resections of parotid SGCs should match the patients’ medical conditions and comorbidities (41). Especially in the elderly, due to the laxity of the skin of the neck, skin defects resulting from the resection of SGCs with cutaneous invasion can be easily resolved by a local rotation flap (e.g. cervicofacial or cervicodeltopectoral flap) with primary closure of the donor site (41, 71). For large skin defects, in elderly patients unfit for free flaps, an island flap based on occipital and posterior auricular perforators or the supraclavicular artery island flap (SCAIF) are good options (41, 72). Also, deep and supporting tissues often need resection. In patients with vasculopathies, pedicled flaps are often preferred for reconstruction over microvascular free flaps (pectoralis major (myo)cutaneous flap, sternocleidomastoid flap, pedicled latissimus dorsi flap) (41, 73). In elderly patients needing a facial nerve reconstruction, static measures for the upper eyelid (gold implant into the upper tarsal plate, lateral canthopexy), nasolabial groove and angle of the mouth (with a tendinous sling) are primordial (41). In a second procedure ptosis can be restored by a brow lift. For dynamic measures, the general adagium that immediate cable grafting with the greater auricular nerve (GAN) yields the best results, does not hold in the same way in the elderly. More reliable solutions for muscle tone conservation are the hypoglossal-facial-jump anastomosis using the GAN, and the increasingly popular “dual nerve transfer” (masseteric nerve to midface division of facial nerve and ansa hypoglossi branch to lower face division), as these nerves lay in the operative field; however, the long term effects – especially in the elderly – still need evaluation (74). Indeed, a functional re-innervation is more difficult to achieve in the elderly population than in younger patients.

## 4 Radiation therapy with photons in the elderly

The role of RT in the management of SGCs mainly consists of postoperative radiation therapy (PORT) initiated after the assessment of pathologic risk factors and within 8 weeks post-surgery (Recommendation 3.7) (17), but for patients with medical comorbidities or unresectable tumors, primary RT with curative intent or – more frequently – with palliative intent are also options (Recommendation 3.10) (17). PORT is recommended for tumors with at least one of the following features, according to the ASCO 2021 (Recommendation 3.2) and the National Comprehensive Cancer Network (NCCN) guidelines v.1.2022 (75):

- intermediate/high grade histology (G2-G3)
- large tumor extension (pT3-T4)
- close or positive resection margins
- neural/perineural invasion
- lymphatic or vascular invasion
- lymph node metastases
- adenoid cystic carcinoma histology

Target volume selection and delineation depend on the location of the primary tumors, the histologic subtype and the neck status. For parotid and submandibular gland tumors, PORT for primary tumor should include the tumor bed with a margin to cover all the surrounding normal tissue in which microscopic tumor infiltration could have spread, such as the para-pharyngeal space for deep parotid tumors, or part of the masseter muscle in case of accessory parotid infiltration. There are no guidelines for mSGCs, and the delineation should be done according to the location of the primary tumor. Several authors published guidelines on target volume delineation for parotid and sub-mandibular glands, and readers are referred to these publications for more detailed information (76–79).

In case of AdCC of the parotid gland, owing to the neurotropism of this histology, delineation of the VII cranial nerve should be done, including the skull base for nerve infiltration close to the mastoid; as AdCC cells may spread along the auriculotemporal nerve sheath, the 3^rd^ branch of trigeminal nerve (mandibular nerve, V3) could also be at risk of infiltration, especially for parotid tumors invading the masticator space. For submandibular gland tumors, the branches of the XII nerve and the lingual nerve (a branch of V3) should be delineated. All patients should be treated with IMRT or VMAT, and a dose of 60 Gy should be delivered in 30 daily fractions of 2 Gy (80, 81). There are no data to justify a higher radiation dose in case of positive resection margins (R1), and the benefit of concomitant chemo-radiotherapy is still unknown. The only randomized controlled trial designed to answer this issue (RTOG 1008) has closed to participant accrual and currently is in the phase of data analysis (NCT01220583). Retrospective data do not support RT implementation with chemotherapy on a routine basis (82), and the most recent ASCO Guidelines do not recommend this strategy (17).

After elective neck dissection, PORT is not recommended for pN0 cases and for patients with a single positive node without extra-nodal spread (ENE-). In cN0 patients who did not undergo neck dissection, a watchful policy is recommended for low-grade and low-stage tumors. Conversely, a prophylactic irradiation of level Ib to IV is advised for high grade tumors, SDC, SCC, adenocarcinoma NOS, and undifferentiated histologies. For patients with multiple positive lymph nodes (N+) and/or for those presenting with extra-nodal extension (ENE+), irrespective of the number of positive nodes, PORT of levels Ib to V is recommended (70, 83).

Guidelines on target volume delineation in the neck have been published (84, 85). Typically, elective dose is delivered in the range of 50 Gy in 2 Gy equivalent, whereas a dose of 60 Gy in 30 daily fractions is recommended for pN+ neck (70). Current data do not justify a higher radiation dose in ENE+ cases, and the benefit of concomitant chemotherapy is unknown. As for the primary tumor bed, intensity-modulated RT (IMRT) or volumetric modulated arc therapy (VMAT) should be used as standard irradiation techniques (86–88). When two different dose levels are used, it is recommended to use a Simultaneous Integrated Boost (SIB) approach.

In patients with unresectable or inoperable tumors due to comorbidities, exclusive RT – either with photons or heavy particles – should be considered as a treatment option. For patients who may not be able to receive curative treatment, palliative RT can improve the quality of life by controlling major loco-regional symptoms such as pain, dysphagia, dyspnea, and bleeding. There is no specific protocol for SGCs, and different RT regimens may be administered, such as 30 Gy in 10 fractions, 20-25 Gy in 5 fractions, or 40-50 Gy in 16 fractions (89). Studies focused on target volume selection, delineation, and dose level as a function of patient age/comorbidities are currently lacking. A study compared a small cohort of 29 elderly patients with major SGCs treated with chemo-radiotherapy or PORT with a matched-pair group of younger patients, without finding any difference in acute or late toxicities (90). In RT for mucosal SCC, age was not reported as a prognostic factor for the development of acute or late toxicity, therefore it is recommended to follow similar guidelines for SGCs RT in elderly as in younger patients (91), also considering that irradiated volumes are typically smaller for SGCs in comparison to other HNCs.

## 5 Particle therapy in the elderly

SGCs of the head and neck are frequently found in proximity to critical structures, such as the paranasal sinuses and the base of skull, and their local control following RT is dose-dependent. Especially in SGCs, the progress of RT techniques throughout the past decades has been achieved by increasing accuracy, and the dose escalation facilitated by high-precision technologies. As opposed to photon RT, proton and heavy ion (carbon) beams exhibit a finite penetration depth within tissue and deposit most of the energy at the end of their path (Bragg Peak), with only minimal dose deposition beyond the Bragg peak. This allows the generation of extremely steep dose gradients resulting in improved sparing of the normal tissue surrounding the target volume. In addition, carbon ions beams generate more complex DNA damage, leading to increased biological effectiveness as compared to either photon or proton RT, and making this technique ideal for SGCs treatment.

High linear energy transfer (LET) RT for SGCs has been explored early on (92, 93). Despite long-term toxicity was significant, the treatment with neutrons achieved a higher local control of disease compared with photons RT, and these early studies led to the investigation of charged particle therapy for SGCs.

### 5.1 Carbon ions (C12)

In 2004, the Chiba group investigators shared their initial experience with carbon ions in HNC. In a prospective pilot trial, patients with various advanced HN malignancies (44% T4) were treated with either an RBE-weighted dose of 52.8-64 Gy of C12 in 16 fractions (4 weeks) or 70.2 GyRBE of C12 in 18 fractions (6 weeks). Despite their unfavorable risk profiles, in patients with AdCC histology carbon ions led to local control rates of 50% at 5 years, with a mild toxicity profile (no G3 toxicity or higher) (94). A follow-up protocol confirmed these results, showing a local control rate of 73% at a median follow-up of 54 months and an OS rate of 47% at 5 years (95). Pooled data of the J-CROS consortium representing four Japanese carbon ion institutions have recently been published (96–101). Koto and colleagues reported outcomes of 458 patients with rare HNC, 27% with AdCC and 4,6% with adenocarcinoma of the nasal and paranasal sinuses, treated with carbon ions between 2003 and 2014. Patients received treatment mostly for advanced disease (overall, 65% Stage cT4) with a variety of C12 treatment protocols with RBE-weighted dose between 57,6 – 60,8Gy in 16 fractions to 65 – 70,2Gy in 26 fractions and normofractionated RBE weighted dose of 70,4Gy. At a median follow-up of 25 months, local control in 122 AdCC patients was 86,5% at 2 years and 77,9% at 2 years in 21 patients with adenocarcinoma (96). In a recent update on 289 patients with AdCC (69% T4), the JCROS working group achieved at 2 years a locoregional control (LRC) rate of 88%, median progression free survival (mPFS) of 68% and a CTCAE G3 late toxicity rate of 15% (98).

The combined treatment of IMRT plus carbon ion boost in active beam application achieved comparable results. An initial analysis based on 29 patients with advanced tumors and gross residual disease, treated either with IMRT+C12 boost (mixed beam) or IMRT (photons only), showed a 4-years LRC of 77.5% and 24.6%, respectively. The OS showed a trend in favor of the mixed beam regimen, but differences at the time were not statistically significant (102). Updated results on 95 patients (94% Stage cT4) confirmed the initial findings: at a median follow-up of 63 months, the 5-years LRC was 60% for mixed beam vs 40% of photons only, and the 5-years OS was 79% vs 60%, respectively. Higher-grade late toxicities remained consistently low (5% G3, no G4-G5 toxicities). No significant LRC differences could be detected between patients following radical resection with gross residual tumor and patients with inoperable disease (103).

The phase II COSMIC trial explored the combination of IMRT and dose escalated carbon ion boost, achieving durable LRC and low toxicity profile (104). Moreover, the comparison of patients treated with this modality following surgical resection and those who had received biopsy only, suggested a more favorable toxicity profile in the latter group, without significant differences in terms of LRC. A retrospective analysis of more than 300 patients with AdCC treated with IMRT plus carbon ion boost from the same institution also confirmed those findings (105). The results were updated and analyzed according to the anatomical site, including a study with 24 patients treated for mixed SGCs of the lacrimal gland and another with 59 patients with nasopharyngeal AdCC (106, 107). In the former study, the LRC was 93% at 2 years; in the latter, focused on inoperable (72%) or incompletely resected AdCC, the LRC was 83% at 2 years, similar to the overall analysis (105). Local recurrence occurred mainly within the gradient to adjacent critical structures; potentially, a harder trade-off may improve results, but also significantly increase high-grade late toxicities (8% G3 AEs).

### 5.2 Protons

Carbon ion facilities are still few, while proton therapy is more accessible. Proton beams have a finite range and Bragg peak, similarly to carbon ions, although their RBE remains similar to photons. Pommier and Linton reported the outcomes of 23 and 26 patients with AdCC treated with standard fractionated protons at 76 Gy (Boston, U.S.A.) (108) and 72 Gy (Indianapolis, U.S.A.) (109). In the former study, 87% of cases had gross residual disease and the 5-years LRC was 95%; in the latter, the 77% of patients had cT4 disease and the 2-years LRC was 92%. Nevertheless, there is a caveat: in the study of Pommier et al., G3 CNS toxicity rate was 43% and two patients developed fatal late effects (9% G5). However, passive beam shaping was used at the time. Gentile et al. treated 14 patients with AdCC of the nasopharynx with 73,8 Gy protons in spot-scanning technique. The cohort included very advanced tumors (90% cT4, 21% with residual disease following debulking and 79% inoperable). Local failures were detected within the high-dose volume, and 5-years OS rate was 59% at a median follow-up of 69 months (110).

The Orsay group reported a series of 13 patients with incompletely resected or inoperable sinonasal AdCC treated with a combination of photons and protons to a median dose of 73,8Gy: the 3-years LRC was 60% and 2-years OS rate 75% at a median follow-up 34 months; 46% of patients experienced G3 ipsilateral hearing loss, due to proximity of tumor to the ear structures (111). The Paul Scherrer Institute group recently shared the outcomes of 35 patients with AdCC treated with protons at a median RBE-weighted dose of 75,6Gy (definitive RT) and 70Gy (post-operative RT): 2-years LRC was 92.2% and OS 88.8% at a median follow-up of 30 months (112).

### 5.3 Re-irradiation with particle therapy

Local relapse following initial RT remains a therapeutic challenge. When salvage surgery is not an option, current systemic treatments only achieve modest response rates and rarely alleviate local symptoms. Re-irradiation is used with caution, however data regarding re-irradiation with carbon ions are emerging. Three groups reported their experience with scanned C12 in HN SGCs:

- In CNAO Pavia (Italy) 51 patients with mixed SGC histologies (74.5% AdCC) were treated within a dose escalation protocol ranging from 15-22 x 3 Gy (RBE weighted dose) to 12 x 5 Gy (RBE weighted dose), with a median RBE-weighted dose of 60 Gy reirradiation dose. At a median follow-up of 19 months, the 1-2 years PFS and OS were 72%-52% and 90%-64%, respectively. No G4 or G5 late toxicities were observed, G3 late toxicities (visual deficits, neuropathy, trismus) was detected in 23% of cases (113).
- In Heidelberg (Germany) 52 patients with AdCC received a median RBE weighted dose of 51Gy and cumulative RBE weighted dose of 128 Gy (3 Gy per fraction) after a median RT-interval of 61 months; with a median follow-up of 14 months, 1-year LRC was 70% and OS rate 88%. Overall response was 54%, while toxicity was moderate (< 7% G3-G4). Following re-irradiation, approximately 35% developed another local relapse within the high-dose area (114); updated results of the Heidelberg group in a mixed cohort of recurrent HNC further support those findings (115).
- In Chiba (Japan) 48 patients with mixed histologies of the HN district (35.4% AdCC) were re-irradiated for local recurrence with a median RBE weighted re-RT dose of 54 Gy (C12) following a median initial RBE weighted dose of 57,6 Gy (C12). At a median follow-up of 27 months, 2-years LRC and OS rate were 41% and 60%, respectively. However, G3 and higher late toxicities reached 38% (116).

Considering the reported results, re-irradiation is feasible, but further dose escalation should be explored with utmost caution. Median age in all the aforementioned cohorts ranged between 43 and 63 years (**Table 5**), and elderly patients constituted a significant percentage of treated patients. Nevertheless, age has been infrequently assessed as a predictor for control and survival rates. Only the large J-CROS series was able to demonstrate a negative impact of higher age on OS (96, 98). Furthermore, radiation therapy on specific subsites may yield a high toxicity impact on elderly patients; Weber et al. showed that age predicted increased visual/ocular toxicity on univariate analysis in a cohort of 36 patients with sinonasal malignancies treated with photons/protons (117).

**Table 5:** Available studies on particle therapy in SGCs and median age of each study population.

First Author	Journal	Year	Particle therapy facility	Histotype	Head and neck subsite	N. of patients	Type of particle therapy	Median age (years)	Influence of age on outcome
**Mizoe** (94)	IJROBP	2004	Chiba, Japan	Mixed	–	36	C12	59-60	–
**Mizoe** (95)	IJROBP	2012	Chiba, Japan	Mixed	–	236	C12	56,5	–
**Koto** (96)	IJROBP	2018	J-CROS, Japan	Mixed	sinonasal	458	C12	63	Age is a prognostic factor for OS
**Shirai** (97)	Cancer Sci	2017	J-CROS, Japan	MEC	–	26	C12	61	–
**Sulaiman** (98)	IJROBP	2018	J-CROS, Japan	AdCC	–	289	C12	58	Age is a prognostic factor for OS
**Hayashi** (99)	Cancer Sci	2018	J-CROS, Japan	Major SGC	–	69	C12	62	No significant influence (multivariate)
**Abe** (100)	Cancer Med	2018	J-CROS, Japan	non-SCC HNC	nasopharynx	43	C12	63	No significant influence (multivariate)
**Hayashi** (101)	Cancer Med	2019	J-CROS, Japan	Mixed	auditory canal	31	C12	55	No significant influence (multivariate)
**Jensen** (103)	Cancer	2015	Heidelberg, Germany	AdCC	–	58	C12+IMRT	58	–
**Jensen** (104)	IJROBP	2015	Heidelberg, Germany	SGCs	–	53	C12+IMRT	58	No significant influence (univariate)
**Jensen** (105)	Radiother Oncol	2015	Heidelberg, Germany	AdCC	–	309	C12+IMRT	56	No significant influence (univariate)
**Akbaba** (106)	Cancer Man Res	2019	Heidelberg, Germany	Mixed	lacrimal gland	24	C12+IMRT	51	–
**Akbaba** (107)	Oral Oncol	2019	Heidelberg, Germany	AdCC	nasopharynx	59	C12+IMRT	59	–
**Pommier** (108)	Arch Otolaryngol Head Neck Surg	2006	MGH, U.S.A.	AdCC	skull base	23	protons	46	–
**Linton** (109)	Head Neck	2015	Indianapolis, U.S.A.	AdCC	–	26	protons	46	No significant influence (multivariate)
**Gentile** (110)	Oral Oncol	2017	MGH, U.S.A.	AdCC	nasopharynx/skull base	14	protons	52	–
**Dautruche** (111)	Cancer Radiother	2018	Orsay, France	AdCC	sinonasal	13	protons +/- photons	55	–
**Pelak** (112)	Oral Oncol	2019	PSI, Switzerland	AdCC	–	35	protons	45,4	Worse prognosis with increasing age
**Weber** (117)	Radiother Oncol	2006	MGH, U.S.A.	Mixed	sinonasal	33	protons	54	Increasing age predics late toxicities (univariate)
**Ikawa** (118)	Cancer Med	2019	Multi-center Japan	non-SCC	oral cavity	76	C12	61,5	–
**Ikawa** (119)	Head Neck	2019	Chiba, Japan	non-SCC	oral cavity	74	C12	56	No significant influence (multivariate)
**Hagiwara** (120)	Head Neck	2019	Chiba, Japan	SGCs	sphenoid sinus	15	C12	55	–
**Hayashi** (121)	Oncotarget	2018	Chiba, Japan	Mixed	lacrimal gland	33	C12	58	–
**Koto** (122)	Head Neck	2017	Chiba, Japan	SGCs	parotid	46	C12	57	–
**Koto** (123)	Head Neck	2016	Chiba, Japan	AdCC	tongue base	18	C12	55	No significant influence (univariate)
**Lang** (124)	Cancers (Basel)	2018	Heidelberg, Germany	AdCC	minor salivary glands	67	C12+IMRT	67	No significant influence (multivariate)
**Morimoto** (125)	Jpn J Clin Oncol	2014	Hyogo, Japan	Mixed	skull base	57	protons or C12	55	–
**Takagi** (126)	Radiother Oncol	2014	Hyogo, Japan	AdCC	–	80	protons or C12	59,5	No significant influence (multivariate)
**Lesueur** (127)	Front Oncol	2019	Orsay, France	AdCC	lacrimal gland	15	protons	43	–
**Hu** (128)	Front Oncol	2020	SPHIC, China	AdCC	sinonasal	38	C12	45	–
**Shirai** (129)	Cancer Sci	2017	Gunma, Japan	non-SCC	–	35	C12	59	No significant influence (multivariate)
**Re-irradiation with particle therapy**									
**Vischioni** (113)	Radiother Oncol	2020	CNAO, Italy	SGCs	re-RT	51	C12	60	Worse prognosis with increasing age
**Jensen** (114)	Radiother Oncol	2015	Heidelberg, Germany	AdCC	re-RT	52	C12	54	–
**Held** (115)	IJROBP	2019	Heidelberg, Germany	AdCC	re-RT	124	C12	NA	No significant influence (multivariate)
**Hayashi** (116)	Radiother Oncol	2019	Chiba/NIRS	Mixed	re-RT	48	C12	56.5	–
**McDonald** (130)	IJROBP	2016	Indianapolis, U.S.A.	Mixed	re-RT	61	protons	62 (SCC) vs 53 (non-SCC)	–

J-CROS, Japan Carbon Ion Radiation Oncology Study Group; MGH, Massachusetts General Hospital, Boston, U.S.A; SPHIC, Shangai Proton Ans Heavy Ion Center; PSI, Paul Scherrer Institut, Villigen, Switzerland; CNAO, National Center for Oncological Hadrontherapy, Pavia, Italy. NA, Not Available.

Despite the treatment of elderly patients is an important issue to address, indicators such as age, quality of life, performance status and comorbidity scores have not been studied in detail so far. Therefore, in this context data are extremely limited. A pooled data analysis of 288 elderly patients with SGCs treated at 3 institutions between 2005 and 2020 was recently performed: 207/288 subjects (72%) were treated with IMRT plus C12 boost, which was correlated with a high LRC (90.6% at 2 years), but the OS was not improved; 70/288 subjects (24.3%) experienced higher grade (G3) acute toxicities, most commonly mucositis, dysphagia and dermatitis. However, toxicities were not reported separately for particle and photon RT, and no structured evaluation of comorbidities was performed (131).

## 6 Systemic treatments: From chemotherapy to precision oncology

Adjuvant systemic treatments are not recommended by the ASCO Guidelines outside of clinical trials, despite the fact that two retrospective studies described a potential benefit of androgen deprivation therapy (ADT) and HER2-blockade in SGCs harboring high-risk pathological features of relapse and overexpression of androgen receptor (AR+) and HER2 receptor, respectively (132, 133). Both studies reported a median age at diagnosis of 60 years (range 29–84 years in the study with ADT; 18–87 years in the study with HER2-blockade) and a prevalence of high-risk histologies, such as SDC. In the former study, 22 patients with stage IVA AR+ SDC received bicalutamide alone or bicalutamide plus LHRHa as adjuvant treatment. At a median follow-up of 20 months, the 3-years disease free survival (DFS) of ADT-treated patients and control group was 48% and 27.7% respectively, with a hazard ratio (HR) of 0.14 (95% CI 0.025 – 0.75, p=0.02). In the latter study on adjuvant HER2-blockade, 34 patients were HER2 positive (1-3+) by immunostaining (41% 3+, 38.2% 2+ and 20.6% 1+). A statistically significant survival benefit of adjuvant trastuzumab versus standard follow-up was observed only in the subgroup with high expression of HER2/neu at immunohistochemistry (IHC 3+ score), showing a DFS of 117 vs 9 months (p = 0.02) and an OS of 74 vs 43 months (p = 0.02). A trial with the antibody-drug conjugate (ADC) ado-trastuzumab emtansine (T-DM1) in the adjuvant setting of HER2-positive SGCs is ongoing (NCT04620187).

Currently, the setting of systemic treatments for SGCs is recurrent/metastatic (R/M) disease without either surgical or (re-)irradiation options. Common sites of distant metastases in SGC are the lung (49–91%), bones (40%), liver (19%), soft tissue (9%), distant nodal basins (8%), brain (7%), kidney (2%), orbit (2%), pancreas (2%) (134).

As specified in the last 2021 ASCO Guidelines, therapy initiation should be considered in the following situations (Recommendation 6.3) (17):

1. symptomatic metastatic deposits not amenable to palliative local therapy;
2. if the growth has the potential to compromise organ function;
3. if lesions have grown more than 20% in the preceding 6 months.

Notably, patients with oligometastatic AdCC and low-grade SGCs with a limited metastatic burden (i.e. ≤ 5 lesions) can be considered amenable to palliative local treatments, either by surgery (e.g. lung metastasectomy) or stereotactic body radiation therapy (SBRT) (Recommendation 6.2) (17). SBRT would be the most suitable option to fragile patients facing a high surgical risk for advanced age and/or comorbidities.

Two single-center studies focused on the setting of advanced AdCC showed that increasing age was related to worse OS: in one study of 105 patients with median age 57.3y (range 19 – 87), the hazard ratio per decade for OS was 1.2 (CI 1.1 – 1.6) (135). In another study of 88 patients with a median age of 58y (26 – 79), the group aged > 60y had worse OS compared with the < 40y group (p=.028) (136). Recently, a prognostic nomogram was proposed as a tool which may help clinicians in recognizing patients with metastatic AdCC who could benefit from a watchful waiting strategy *vs* active treatment (137). The study included 298 patients with a median age of 51y (range 42 – 60) in the testing cohort (n=259) and 59y (range 48 – 68) in the validation cohort (n=39). The nomogram is based on five independent prognostic factors (gender, disease-free interval and presence of lung, liver or bone metastases) that can predict the overall survival at 3, 5 and 7 years. Therefore, age was not confirmed as an independent prognostic factor for OS in this group of R/M AdCC patients.

### 6.1 Chemotherapy

Few clinical trials investigated the efficacy of cytotoxic chemotherapies in patients with R/M SGCs. In most of the studies, the population number was less than 40 patients and included a variety of histotypes, both AdCC and non-AdCC (**Table 6**). Consequently, there is not a standard-of-care regimen for SGCs, and the choice of treatment is often directed by the toxicity profile of drugs that proved an activity in this setting.

**Table 6:** Poli-chemotherapy regimens for SGCs and median age of study populations.

First Author	Year	N. pts and histologies	Combination regimen	Median age (range)	ORR (%)	Duration of response and PFS
**Licitra** (138) Phase II trial	1996	22 pts AdCC = 12 Non-AdCC = 10	**CAP** regimen (d1 q21): • **Cyclophosphamide** 500 mg/sqm bolus i.v. • **Adriamycin** 50 mg/sqm • **Cisplatin** 50 mg/sqm in 30 minutes up to PD/toxicities	50 (33 – 65)	29% (95% CI 11-50%) PR = 27% CR = 0 Mean number of cycles = 4 (2-6)	DoR = 7 months (3 – 13)
**Airoldi** (139) Prospective study	2000	14 pts AdCC = 10 Non-AdCC = 4	• **Carboplatin** AUC 5.5 q21 • **Paclitaxel** 175 mg/sqm q21	55 (20 – 70)	**14%**	DoR = 8.5 months (5 – 12)
**Airoldi** (140) Phase II trial	2001	16 pts AdCC = 9 Non-AdCC = 7	• **Cisplatin** 80 mg/sqm q21 • **Vinorelbine** 25 mg/sqm dd1,8 q21 up to 6 cycles	58 (20 – 68)	**34%** PR = 25% CR = 19%	median duration of PR = 7.5 months (3 – 11) median duration of CR = 15 months (6-27+)
**Laurie** (141) Phase II trial	2010	33 pts AdCC = 10 Non-AdCC = 23	• **Platinum** • (either cisplatin 80 mg/sqm d1 or carboplatin AUC 5 d1 q21) • **Gemcitabine** 1000 mg/sqm d1,8 q21 up to 6 cycles	58 (33 – 81)	**24%** (95% CI 11-42%) PR = 21% CR = 3% *OR were observed in patients who received cisplatin.*	DoR = 6.7 months (1.3 – 11.3)
**Nakano** (142) Retrospective study	2016	38 pts AdCC = 9 SDC = 18 Other SGC = 11	• **Carboplatin** AUC 6 q21 • **Paclitaxel** 200 mg/sqm q21	60 (29 – 75)	**39%** overall **9%** in AdCC subgroup	mPFS = 6.5 months
**Okada** (143) Retrospective study	2019	24 pts SDC = 12 Non-SDC=12	• **Carboplatin** AUC 5 • **Docetaxel** 70 mg/sqm	58 (37 – 55)	**42%**	mPFS = 8.4 months

AdCC, adenoid cystic carcinoma; AUC, Area Under the Curve (mg/ml/s); ORR, objective response rate; PD, progressive disease; PR, partial response; CR, complete response; mPFS, median progression free survival.

According to 2021 ASCO Guidelines, symptomatic patients with R/M SGCs requiring a rapid objective response may be offered platinum-based chemotherapy combinations. Solid data on the tolerability of these regimens by the elderly population are lacking, therefore they should be offered only to fit patients. In the aforementioned studies, the median age of the population was below 65, and the majority employed cisplatin as backbone agent. However, cisplatin is rarely an option for elderly patients, due to the frequent comorbidities (e.g. renal function impairment) and the drug-related risk of nephrotoxicity and ototoxicity, which may impact on the quality of life. The replacement of cisplatin with carboplatin is generally considered less effective (141). The only regimens that proved a long-term clinical benefit with carboplatin were the associations with taxanes (139, 142). In the latter study by Nakano et al., 38 SGCs patients (median age 60y) received carboplatin (AUC 6 q21) and paclitaxel (200 mg/sqm q21), reporting G3/4 neutropenia (53%), anemia (13%) and thrombocytopenia (13%). In another study, the association of carboplatin (AUC 5) and docetaxel (70 mg/sqm) provided an ORR of 42%, with a manageable safety profile (G3/4 neutropenia and anemia observed in 20-30% of the patients) (143).

Single-agent cisplatin 100 mg/sqm q21 for 4 cycles (144), vinorelbine (VNB) 30 mg/sqm i.v. weekly (140), and paclitaxel 200 mg/sqm q21 (ECOG 1394 study) (145) provided an objective response rate (ORR) of 18%, 20% and 16%, respectively.

The choice of chemotherapy should be based on the patient’s characteristics (e.g. performace status, comorbidities, biological age) and tumor histotype. Before starting any chemotherapeutic agent, it is recommended to perform:

- a detailed clinical history in order to investigate the comorbidities that would impact on the choice of chemotherapy (e.g. doxorubicin is discouraged in case of cardiac dysfunction, taxanes in case of diabetic neuropathy or chronic liver disease, cisplatin in case of kidney impairment);
- a G8 questionnaire, in order to identify the frail patients who would benefit from a comprehensive geriatric assessment and a de-escalated treatment;
- a complete blood test including blood count, renal and liver function;
- a baseline electrocardiogram with QTc interval.

Carboplatin and taxane combinations may be offered to fit elderly patients with non-AdCC histotypes (e.g. adenocarcinoma, NOS; SDC) requiring a tumor shrinkage. However, a careful monitoring of the complete blood count should be carried out to check the risk of treatment-related leukopenia or thrombocytopenia. Conversely, elderly patients at risk of renal insufficiency and/or taxane-related peripheral sensory neuropathy, due to concomitant comorbidities (e.g. diabetes, vasculopathy) (146), should receive less neurotoxic chemotherapy combinations, or single-agent vinorelbine.

### 6.2 Androgen deprivation therapy for androgen receptor expressing SGCs

The androgen receptor (AR) is a steroid hormone receptor activated by testosterone and the more potent dihydrotestosterone (DHT) ligands. The receptor-ligand engagement in the cytoplasm induces the AR homodimerization and translocation of the complex in the nucleus, where it acts as a transcription factor. AR is not expressed in normal salivary glands, but it is overexpressed most frequently in SDC (86%) and carcinoma ex-PA (90%). It has been found less commonly in adenocarcinoma NOS (26%), AcCC (15%), MEC (5%) and AdCC (5%). AR positivity in epithelial-myoepithelial carcinoma is anecdotal, mostly reported in the apocrine variant (147). AR-expressing (AR+) SDC, adenocarcinoma NOS and carcinoma ex-PA are mostly found in elderly male patients, supporting the hypothesis of a hormonal-dependence pathogenesis of these tumors. Of note, these histotypes harbor a significant quote of overlapping cases, as almost 40-50% of SDCs arise from carcinomas ex-PA (148).

As shown in prostate cancers, the androgen axis can be targeted at different levels:

1. Pituitary gland: gonadotropin-releasing hormone (GnRH) receptor agonists, specifically LHRH analogs, that are the backbone of androgen-deprivation therapy (ADT) since they inhibit the synthesis of androgens;
2. Adrenal glands: abiraterone inhibits the conversion of testosterone to DHT by blocking the steroid 5α-reductase 1 membrane-bound enzyme;
3. Intracellular: bicalutamide and enzalutamide compete with androgens for the binding to AR, inactivating the receptor.

The association of bicalutamide and LHRH analog is known as combined androgen blockade and ADT. ADT is included by the 2021 ASCO Guidelines among the options for first- or subsequent-line setting in non-AdCC AR+ SGCs (Recommendation 6.5), on the basis of a single-arm phase II trial of leuprorelin and bicalutamide in 36 patients with AR+ R/M SGCs showing an objective response rate (ORR) of 42% (11% CR) and a median PFS of 8.8 months (149). The median age of this study population was 67 years (range 46–90), 22% with > 75 years, and only 14% had received ChT in the first line. Therefore, ADT could be a feasible chemo-free first-line therapy for elderly patients with AR+ SGCs. A single institution retrospective cohort study on 58 pts with SDC or high-grade adenocarcinoma NOS treated in the first line either with ADT or ChT, reported that OS was comparable in the two groups, but response rates to first-line ADT were higher than those with ChT (150). However, the results of the currently ongoing EORTC 1206 randomized clinical trial are expected to answer the question whether ADT is better than ChT as first-line therapy for AR^+^ SGCs (NCT01969578).

A phase II trial of the second-generation antiandrogen enzalutamide administered as single-agent in a cohort of previously treated and ADT–naive AR^+^ SGC patients, reported an ORR of 4% (151). Recently, a phase II study tested the efficacy of abiraterone plus ADT in 24 patients with AR+ R/M SGCs who progressed on ADT, reporting an ORR of 21% with a mPFS of 3.65 months (95% CI, 1.94 to 5.89). Fatigue was the most frequent G3 treatment-related adverse event (AE), reported by 8% of patients (152). The median age of this study population was 65.8 years (range 44 – 77). This trial paves the way to a possible anti-androgen treatment sequence in AR+ SGCs.

The clinical benefit from ADT seems to be higher for those cases with AR immunostaining showing strong intensity and diffusion (≥ 70% of positive nuclei) (153), and with high AR pathway activity (154). However, different mechanisms of resistance have been described, from crosstalk with other pathways (HER2, glucocorticoid receptors) to alternative isoforms of AR lacking the ligand-binding domain, and AR-independent activation of the proliferative transcription program (155). Of note, almost 35–50% of SDCs present the ARv7 isoform, detected by IHC also in 15% of ADT-naïve patients (156). In future studies, liquid biopsies could help to investigate the role of AR-v7 as primary/secondary resistance mechanism to ADT in AR+ SGCs, as previously done in prostate cancer (157, 158). Some cases with chemo-hormonal combinations have been reported (159), but it is still to be defined whether there is an extra benefit of the combination over the single components.

### 6.3 Targeted therapy

#### 6.3.1 HER2-blockade

The *ERBB2* gene codifies for the human epidermal growth factor receptor 2/neu (HER2/neu). HER2 expression can be detected by IHC, with a scoring reported as negative (0/1+), equivocal (2+) or positive (3+), and/or by *in situ* hybridization (ISH). It is overexpressed (IHC 3+ or IHC 2+ with positive ISH) in almost 8% of SGC cases (160), with the highest prevalence in high-grade subtypes, as it can be found in 43% of SDCs, 39% of carcinoma ex-PA, 17% of SCC and 13% of adenocarcinomas, NOS (161). Conversely, AdCCs express low levels of HER2, as described in a phase II study of 20 patients where 30% had HER2 IHC 1+, 5% IHC 2+ and 0% IHC 3+ score (162).

According to the 2021 ASCO Guidelines, patients with HER2-positive SGCs may receive HER2-blockade as first- or subsequent-line therapy (Recommendation 6.5). Many agents have been tested in the last years and are already approved for the treatment of R/M HER2-positive breast cancer. In **Table 7** are summarized the studies that explored HER2-blockade in SGCs.

**Table 7:** Studies that explored HER2-blockade in SGCs and median age of study populations.

First Author	Year	N. pts	HER2-blockade	Median age (range)	ORR (%)	Survival outcomes
**Agulnik** (162) Phase II trial	2007	**20** ACC **19** non-ACC	**Lapatinib** 1500 mg daily	52 (38 – 72) 64 (45 – 80)	0 (36% SD)	NA
**Limaye** (163) Retrospective study	2013	**5** SDC HER2 3+	TCH scheme: • Trastuzumab* • **Carboplatin** AUC 5-6 q21 • **Paclitaxel** 175 mg/sqm q21 ChT given up to 6 cycles and trastuzumab until PD	63 (51 – 82)	**100%** n.4 PR n.1 CR (at 15 months)	DoR=8–18 months Patient with CR withdrawed trastuzumab after 2 years (NED) and started active surveillance
**Takahashi** (164) Phase II trial	2019	**57** Prior systemic therapy allowed	• Trastuzumab* • **Docetaxel** 70 mg/sqm q21	57 (38 – 82)	**70%** 14% CR 32% PR	mPFS=9 months
**Kurzrock** (165) Phase IIa basket trial MYPATHWAY (NCT02091141)	2020	**15** SGCs Prior systemic therapy allowed	• Trastuzumab* • Pertuzumab**	59 (37 – 80)	**60%** n.8 PR n.1 CR	mDoR=9.2 months mPFS=8.6 mos
**Swed** (166) Retrospective study Single institution	2019	**7** (6/7 = 86% pretreated with trastuzumab)	T-DM1***	58 (45 – 67)	[86%] n.3 CR (43%) n.3 PR (43%) n.1 mixed	4/6 (67%) had a duration of treatment > 10 months
**Li** (167) Phase II basket trial (NCT02675829)	2019	**10** Pretreated with HER2-blockade (Trastuzumab, Pertuzumab)	T-DM1***	65 (36 – 90)	**90%** n.5 CR after prior HER2-blockade	mDoR and mPFS not reached at a median FUP of 12 months (range 4 – 20)
		*HER2 amplification was identified by NGS and tumors were subsequently tested by FISH and IHC. HER2 amplification by NGS (fold change 2.8 to 22.8) correlated with HER2/CEP17 ≥ 2 by FISH (8/8 tested) or IHC 3+ (10/10 tested).*				
**Jhaveri** (168) Phase II trial NCI-MATCH subprotocol Q	2019	**3** Prior systemic therapy allowed (no prior HER2 blockade)	T-DM1***	NR 64 (39 – 80) referred to the trial cohort	[67%] n.2 PR	6-months PFS = 23.6% DoR=24 months for a SCC of the parotid and 9 months for a MEC of the parotid
		*Eligible patients had HER2 amplification at a copy number (CN) >7 based on targeted NGS with a custom Oncomine AmpliSeq™ (ThermoFisher Scientific) panel.*				
**Tsurutani** (169) Phase I study	2020	**8** Pretreated with HER2-blockade	**T-DXd** Tested in 3 groups: non-small cell lung cancers, colorectal cancers and other cancers (including n.8 SGCs)	NR 58 (44 – 76) referred to the group of other cancers	SGCs among the histologies with promising tumor shrinkage	mDoR not reached (95% CI, 3.0–not reached). mPFS = 11.0 months (95% CI, 2.8–NE) referred to the group of other cancers

*Trastuzumab standard dose: loading dose of 8 mg/kg i.v. followed by 6 mg/kg i.v. every 3 weeks; **Pertuzumab 840 mg i.v. loading dose, followed by 420 mg i.v. every 3 weeks (q3w); ***T-DM1 = Ado-trastuzumab emtansine, 3.6 mg/kg i.v. every 3 weeks; T-DXd, Trastuzumab-deruxtecan; MEC, mucoepidermoid carcinoma; SCC, squamous cell carcinoma; NR, not reported. NA, Not Available.

In a trial with docetaxel-trastuzumab (164) the median age of study population was 57 years (range 38-82), with 14% of patients older than 75 years; 14% of patients reported G3 febrile neutropenia, and 60% have at least one episode of G4 neutropenia, making this regimen feasible only for selected elderly patients. In a retrospective trial with the triplet of carboplatin-paclitaxel-trastuzumab, G3 fatigue and thrombocytopenia were seen in one patient each, no G4-G5 toxicities occurred and no cardiac dysfunction was seen as a result of trastuzumab-based therapy. In this small retrospective study two elderly patients were included (72 and 82 years), and both managed to receive at least 8 months of trastuzumab-taxane scheme (163).

Dual blockade with trastuzumab and pertuzumab (anti-HER2-HER3 dimerization) was tested in a phase II basket trial with 15 patients that had been previously treated with chemotherapy (patients with active brain metastases were excluded) and showed an ORR 60% with durable responses (165).

The antibody drug conjugate (ADC) Ado-trastuzumab emtansine (T-DM1) is formed by a molecule of trastuzumab linked to 3–4 molecules of the maytansine derivative DM1, a tubuline inhibitor. T-DM1 was investigated in two basket trials where the SGC subgroup showed high objective response rates, up to 90% in the most successful one, that also included patients pretreated with HER2-blockade who achieved 50% of complete responses after prior trastuzumab, pertuzumab and androgen-deprivation therapy (167). The NCI-MATCH trial-subprotocol Q did not meet its primary endpoint of ORR > 16% in a heavily pretreated population composed by various histologies; nevertheless, the authors warranted further studies of T-DM1 in SGCs, as 2 out 3 patients with SGCs enrolled in this trial achieved durable responses (168).

The ADC trastuzumab deruxtecan (T-DXd or DS-8201a) is formed by a molecule of trastuzumab conjugated with a topoisomerase I inhibitor payload (deruxtecan). It is currently approved in HER2-positive R/M metastatic breast cancer, pretreated with HER2-blockade. In a phase I study, HER2-amplified SDCs were among the histologies with the most pronounced tumor shrinkage, although topoisomerase inhibitors are not part of the cytotoxic agents commonly used in SGCs (169).

Globally, anti-HER2 agents provide objective and durable responses in HER2-overexpressing SGCs. Also, no specific adverse events have been related with advanced age, therefore they are potential chemo-free option for elderly patients. Despite the recently observed increase in anti-HER2 agents, there is still a therapeutic void for patients with HER2-positive SGCs. Trials are urgently needed to select the best sequencing of anti-HER2 strategies, as done in breast cancer, and also to define the optimal strategy for the patients with AR and HER2 co-expression, which display a short overall survival (170).

#### 6.3.2 Multikinase inhibitors and single-targeted therapies

AdCC is associated with low responses to chemotherapy. However, AdCC may harbor hyperactivation of the angiogenic pathway, as demonstrated by recent in-depth analysis of the mutational landscape of recurrent or metastatic (R/M) AdCC (171). According to the 2021 ASCO guidelines, patients who are candidate to start systemic therapy, can receive multikinase inhibitors (MKIs) instead of ChT, on the basis of phase II trials proving the efficacy of lenvatinib and sorafenib (Recommendation 6.4) (17); the median age of study populations was 57y (range 38–73) and 50y (range 21–70), respectively (172, 173). Currently, lenvatinib is the most frequently used MKI for R/M AdCC; it is a second-generation MKI directed to the targets of vascular endothelial growth factor 1-3 (VEGF1–3), fibroblast growth factor receptors 1-2 (FGFR1–4), RET, c-KIT, stem cell growth factor receptor (SCGFR) and platelet-derived growth factor receptor α (PDGFRα). However, up to 50% of patients treated with lenvatinib may experience G3 adverse events, global functioning impairment and increasing fatigue, especially across the first 6 months of treatment (174). Recently, a randomized phase II trial in R/M AdCC showed that the selective VEGFR1–3 inhibitor axitinib, as compared with observation, with a median follow-up of 25.4 months significantly improved the 6-month PFS (73% vs 23%). Even though the ORR of axitinib was 0%, median OS was not reached with axitinib vs 27.2 months in the observation arm (p = 0.226). The cohort treated with axitinib included 30 patients with a median age of 57y (range 28 – 77), and the most frequently reported adverse events for axitinib were oral mucositis and fatigue (175). Recently, at the ASCO 2022 Annual Meeting, a phase II study evaluated the efficacy and safety of the selective VEGFR2-inhibitor rivoceranib (700 mg once daily) in R/M AdCC (Kang H et al., abstract 6020 – NCT04119453). The study was conducted on a population of 80 patients with median age of 54.5y (range 28-76). At a median follow-up of 15 months, ORR was 13.9% in patients pretreated with VEGFR-TKI and 16.9% in VEGFR-TKI naïve; median PFS was 9.2 months regardless of prior anti-VEGFR treatment. 80% of the population experienced at least one G3 AE, mostly hypertension, stomatitis, anemia and fatigue. In the clinical practice, special attention should be addressed to elderly patients with comorbidities such as hypertension and impaired kidney function treated with antiangiogenic agents, as they require a full basal cardiological evaluation with echocardiogram and strict monitoring of blood pressure and proteinuria throughout the treatment.

AdCCs may harbor mutations in genes encoding chromatin-state regulators, *TERT* promoter, fibroblast growth factor (FGF)–insulin-like growth factor (IGF)–PI3K pathway and *RET* (4%) (171, 176). The *MYB-NFIB* fusion is the diagnostic hallmark of AdCC, being present in 65 – 80% of cases, but it is not currently actionable by approved drugs. However, the ionophoric antibiotic monensin has been recently identified as a potent *MYB* inhibitor in preclinical studies (177), and a phase I trial with TetMYB vaccine and immunotherapy (anti-PD1 agent BGB-A317) has recently completed the accrual (NCT03287427). *NOTCH* mutation is found in 10-22% of AdCC and identifies a subgroup of AdCCs prone to develop liver and bone metastases, leading to a dismal prognosis (178) especially when the *MYB* fusion is co-present (171). The phase II trial ACCURACY showed the clinical activity of pan-Notch inhibitor AL101 at 4 mg once weekly (QW), achieving a disease control rate of 68% (15% partial responses) in a cohort of 45 patients with median age of 50 years (179). At the ESMO 2021 Congress, the results of 6 mg QW cohort were presented, showing good tolerability of AL101 in a study population of 37 patients with median age of 59 years (180).

In non-AdCC SGCs, the use of MKIs is not recommended (181). However, in certain cases, single molecular targets can be addressed. *NTRK* gene fusions, codifying for oncogenic forms of tropomyosin receptor kinase (TRK), occur in an estimated 5% of cases of SGCs, including more than 90% of secretory carcinomas (SC). In the majority of cases, SC presents as an indolent nodule arising from the parotid and harbors the chromosomal translocation *t(12,15)* between the *ETV6* gene on chromosome 12 with *NTRK3* on chromosome 15, generating the fusion product *ETV6*–*NTRK3* (33). The nuclear pattern of pan-TRK IHC staining has good sensitivity to detect the *ETV6–NTRK3* fusion, helping in the differential diagnosis. From the histological prospective, secretory carcinoma could be misdiagnosed most frequently with AcCC, but also with AdCC, cystadenocarcinoma, MEC and low-grade carcinoma NOS. Recently, the efficacy of TRK-inhibitor larotrectinib for R/M SGCs has been analyzed in a study where the investigators focused on the population of two clinical trials: the phase 2 NAVIGATE basket trial (NCT02576431) and a phase 1 trial (NCT02122913). Overall, 24 patients were treated with 100 mg larotrectinib twice daily and 1 patient received 150 mg twice daily. The median age of study population was 58.5 years (range 28 – 78). Tumor histology was SC in 54%, adenocarcinoma in 21%, and MEC in 13%, followed by AdCC, glandular sarcomatoid carcinoma, and adenocarcinoma NOS. All patients had an *ETV6-NTRK3* gene fusion. The ORR to larotrectinib was 92% (95% CI, 73% – 99%), with 79% PR and 13% CR. The median time to response was 1.84 months (range 0.99 – 5.98) and the duration of treatment ranged from 0.95 to > 60.4 months, with a 36-months PFS rate of 66% and OS rate 91% (182). From the integrated analysis of three phase I and II clinical trials on entrectinib (ALKA-372-001, STARTRK-1, and STARTRK-2), a response to this drug was seen also in 6 out 7 patients with SC, with an ORR of 86% (183). Both larotrectinib and entrectinib received FDA and EMA tissue-agnostic approval, and ASCO 2021 Guidelines on SGCs recommend the use of TRK-inhibitors in first or subsequent-line, rather than chemotherapy (Recommendation 6.5) (17). However, different mechanisms of acquired resistance to TRK-inhibitors have been described, either on-target mutations in the drug-binding site, or off-target activation of the mitogen-activated protein kinase (MAPK) pathway (184–186). Currently, clinical trials with next-generation TRK-inhibitors selitrectinib (NCT03215511) and repotrectinib (NCT03093116) are ongoing (187). Of note, a case of entrectinib-resistant SC that achieved a durable response to selitrectinib also on leptomeningeal metastases was recently reported (188).

Few cases of SC with high-grade histology and aggressive behavior have been described in association with *ETV6-MET*, *ETV6-RET* and *VIM-RET* fusions, which are not detectable by pan-TRK IHC and could be actionable by other targeted therapies, especially RET inhibitors in the case of *ETV6-RET* fusions (34–36). Therefore, all tumors with morphological features resembling secretory carcinoma should undergo Next Generation Sequencing in order to detect the specific fusion transcripts, as recommended by the ESMO guidelines on molecular profiling of solid tumors (189, 190).

In the last decade several studies reported single-gene potential targets with different incidence, stratified by histotypes and grade, and uncovered novel therapeutic targets by comprehensive molecular profiling (27, 191–193). For patients who may be potential candidates for systemic therapy and have SGC histologies with unknown driver mutation status, the ASCO 2021 Guidelines on SGCs recommend performing a comprehensive panel for driver mutations by NGS profiling (Recommendation 6.9) (17). Currently, mutations in *RET, BRAF, HRAS, FGFR, PIK3CA, BRCA1/2, PTCH1* genes are actionable by oral drugs already approved for other cancer types, and basket clinical trials/managed access programs are currently ongoing. This is a promising area of research also for elderly patients, usually more fragile and difficult to treat with standard chemotherapy.

### 6.4 Immunotherapy

The microenvironment of SGCs is highly heterogeneous, ranging from the immune-exclusion of AdCC to the high immune-infiltration and high PD-L1 expression of high-grade histologies, such as carcinoma ex-PA (75%), MEC (57.1%) and SDC (50%) (194–196).

Currently, the role of immune checkpoint inhibitors (ICI) in SGCs is a subject of active research (**Table 8**). According to the ASCO 2021 Guidelines (17), ICI should be offered for patients with high tumor mutational burden (TMB) or high microsatellite instability (MSI-H). Also, the NCCN guidelines (v.1 2022) limit the use of pembrolizumab to the cases with TMB-high, on the basis of the KEYNOTE-158 study that proved the agnostic efficacy of pembrolizumab in solid tumors with TMB ≥ 10 mutations identified per megabase (202). Of note, the cohort J of KEYNOTE-158 dedicated to SGCs histology included 3 patients with TMB-high and 79 patients with TMB-low; one SGC tumor was found MSI-high. Objective responses to pembrolizumab were reported in 1/3 cases of TMB-high and in 3/79 of TMB-low. Therefore, TMB-high did not show a high sensitivity as predictive biomarker of response to anti-PD1 inhibition in SGCs. Currently, the tumor-agnostic approval of pembrolizumab upon TMB-high status has not been endorsed by the European Medical Agency.

**Table 8:** Studies that explored the efficacy of immunotherapy in SGCs and median age of study populations.

First Author	Year	N. pts	Immunotherapy	Median age (range)	ORR (%)	Survival outcomes
**Cohen** (197) Phase Ib trial KEYNOTE-028	2018	26 AdCC and non-AdCC* PD-L1 expression on ≥1% of tumor or stroma cells required	**Pembrolizumab** 10 mg/kg i.v. every 2 weeks	57 (23 – 72)	12% n. 3 PR**	mPFS 4 months (95% CI: 2-5 months) mOS 13 months (95% CI 6 – NR)
		*Histotypes: adenocarcinoma NOS (38%), mucoepidermoid (12%), undifferentiated (8%), squamous cell (8%), and AdCC (8%). **PR observed in two adenocarcinoma NOS and in one high-grade serous carcinoma.				
**Fayette** (198) Phase II trial NISCAHN	2019	52 Non-AdCC 46 AdCC	**Nivolumab** 3 mg/kg IV every 2 weeks up to 12 months	61 (29 – 81)	Non-AdCC: n.2 PR (3.8%) n.22 SD (42.3%) AdCC: n.4 PR (8.7%) n.26 SD (56.5%)	Non-progression rate at 6 months: 14% non-AdCC 33.3% AdCC
**Rodriguez** (199) Phase I/II trial	2020	25 n.12 AdCC n.3 AcCC n.3 MEC	• **Vorinostat** (HDAC) 400 mg given orally 5 days on and 2 days off during each 21-day cycle • **Pembrolizumab** 200 mg q21	61 (33 – 86)	16% (95% CI 5% - 37%) n.4 PR*** n.14 SD (10 SD > 6 months)	Median duration of treatment = 24 weeks; mDoR=10.5 months (8.7–21) mPFS 6.9 months mOS 14 months
		***PR observed in one patient with lymphoepithelioma-like carcinoma of the parotid, two patients with acinic cell carcinoma, and one patient with adenoid cystic carcinoma.				
**Burman** (200) Phase II trial (NCT03172624)	2021	32 Non-AdCC	• **Nivolumab** 3 mg/kg q2 weeks • **Ipilimumab** 1 mg/kg q6 weeks until PD or intolerant toxicity	64.5 (30 – 87)	16% overall 25% in the SDC group (profound responses)	Duration of therapy from 15.7 to 29.5 months
**Ferrarotto** (201) Phase II trial NCT03990571	2022	28 AdCC	• **Axitinib** 5 mg po bid • **Avelumab** 10 mg/kg iv q2 weeks	55 (29 – 88)	17.5%	mPFS = 7.2 months (95% CI: 3.7-11.7) 6-mos PFS rate = 57% (95% CI_41-79%). mOS=17.4 months (95%CI:13-NA) mDOR=5.2 mos (95% CI: 3.7-NA)

HDAC, histone deacetylase inhibitors.

At the 2021 ASCO Annual Meeting, the results of Cohort 2 of NCT03172624 study were reported (200). This phase II trial included 32 patients affected by R/M non-AdCC SGCs, who received anti-PD1 nivolumab plus anti-CTLA4 ipilimumab. The majority of patients were diagnosed with SDC (38%) and AcCC (22%) histotypes. Median age was 64.5 (range 30 – 87); 69% of the total population had received a prior chemotherapy, 22% androgen-deprivation therapy and 25% other targeted therapies (no ICI). The study met the primary endpoint of best overall response with 5 responses (16%) in the overall population, while the SDC subgroup showed a PR rate of 25% (3/12). The 5 confirmed responders had regressions ranging from -66% to -100% in target lesions, with a duration of therapy ranging from 15.7 to 29.5 months. Of note, the RNAseq analysis highlighted a correlation between baseline immune infiltration and benefit from ICI. In a recent case series of Chinese patients with R/M AdCC treated with immunotherapy, the response to PD-1 checkpoint inhibitors (camrelizumab or pembrolizumab) was related to elevated T-cell infiltration score and antigen-presenting machinery score (203). Recently, the immunomodulatory role of antiangiogenic drugs has been explored. At the 2022 ASCO Annual Meeting, the results of a phase II clinical trial of VEGFR1-3 inhibitor axitinib (5 mg BID) associated with PD-L1 inhibitor avelumab (10 mg/kg every 2 weeks) in patients with R/M AdCC were presented: of 28 patients with evaluable treatment efficacy, 16 had received the treatment in first line. Median age of study population was 58y (range 29 – 88), 39% had AdCC with solid component, 36% with cribriform/tubular histology. The ORR was 14.3% (4/28, 95%CI: 4-32.7%) and responses were independent from NOTCH1 activating mutation; 6-months PFS rate was 57%. The most common treatment-related adverse events were fatigue, hypertension and diarrhea (Ferrarotto R et al., Abstract #6019 - NCT03990571).

The most relevant trials that explored the activity of immunotherapy in SGCs are resumed in **Table 8**. Overall, the efficacy of immunotherapy is still under investigation in SGCs, but in a subset of patients it provides profound and durable responses. Reliable predictive biomarkers beyond TMB are needed, in order to select the population of patients who could benefit the most from this type of therapy usually well-tolerated also by elderly patients.

## 7 Discussion

Elderly patients with SGC carry a poor prognosis compared to younger patients. We have reported the incidence and the 5-years survival outcomes of the most common histotypes in the U.S. population, in order to assess whether a different distribution of histotypes in the two main age subgroups (< 65y and 65y+) could explain part of this survival disparity. Good prognosis histotypes and early stage at diagnosis were more common in younger patients. Conversely, advanced stage at diagnosis and unspecified (NOS) histotypes presented more frequently in the elderly. These characteristics are possibly related to the aggressiveness of disease, poor attention to early symptoms, and difficulty in accessing referral hospitals to receive an accurate diagnosis and appropriate treatment. Such features, typically found in elderly, have been recognized as independent poor prognostic factors in SGC patients, regardless of the type of treatment (17, 18). Also, the high proportion of “carcinoma, NOS” in the elderly group could be an indirect sign of suboptimal pathological diagnosis in this population.

Since a correct pathological diagnosis is the prerequisite for an appropriate treatment planning, proper pathological and molecular analyses should be carried out, both for diagnostic and therapeutic purposes, in order to avoid the generic diagnosis of “carcinoma, NOS”. As assessed in the ASCO 2021 Guidelines (Recommendation 1.6), the use of ultrasound-guided core needle biopsy (CNB) has an estimated sensitivity of 94% and specificity of 98% (204). Moreover, CNB has a lower inadequacy rate (1.2%) than FNAB (8%) and yields adequate material for ancillary molecular testing (205, 206).

**Figure 2:**
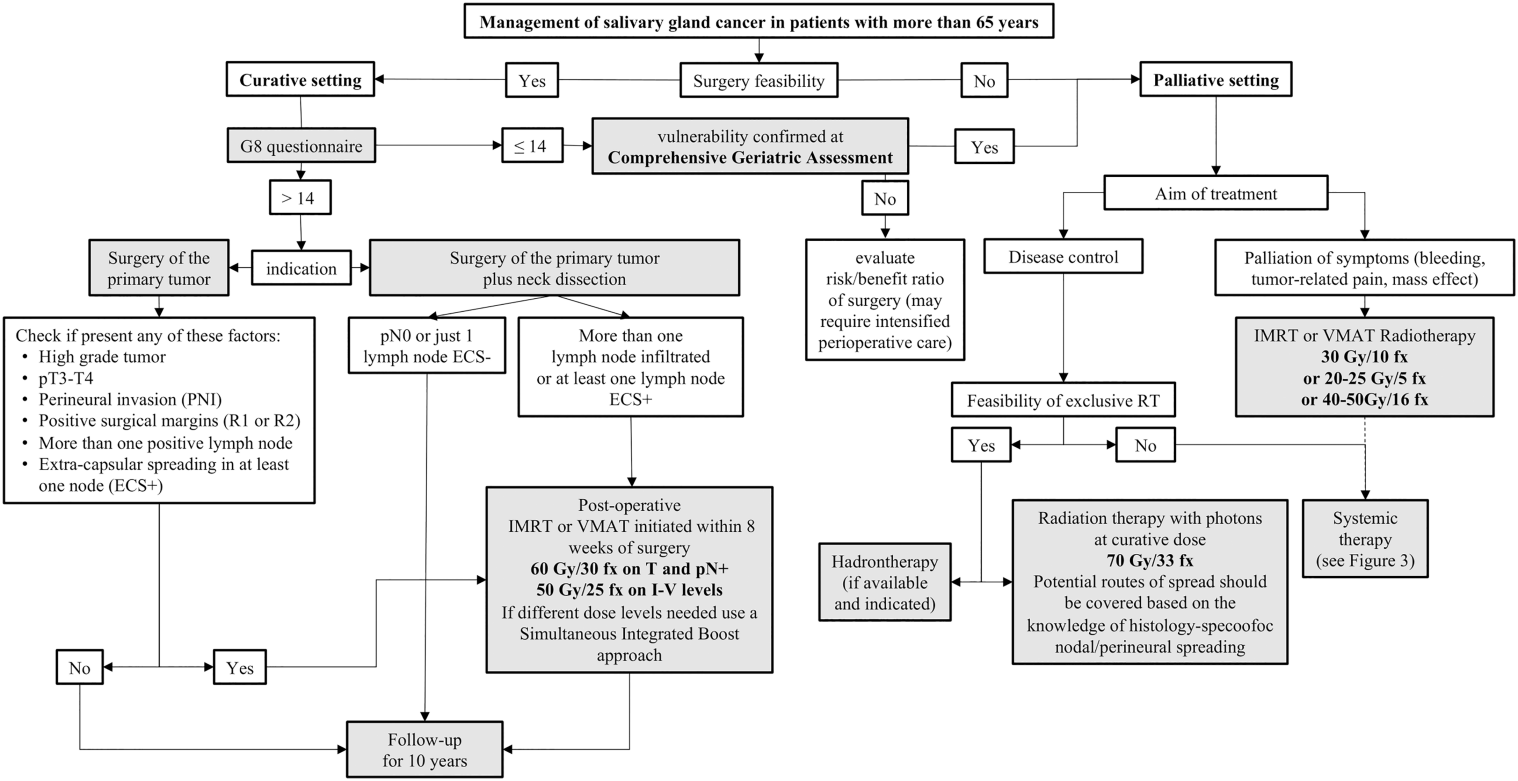
Algorithm proposal for the clinical assessment and treatment strategies of elderly patients with SGCs. G-8,; PNI, perineural invasion; R1, microscopic residual tumor; R2, macroscopic residual tumor; ECS, extra-capsular spreading (+ present, - absent); Gy gray; fx fractions; T, tumor; N, lymph nodes; IMRT Intensity-modulated radiation therapy; V-MAT, Volumetric modulated arc therapy; IHC, immunohistochemistry; SC, secretory carcinoma; NGS, next generation sequencing; TMB, tumor mutational burden; BSC, best supportive care.

In Europe, the centralization for HNCs cases is feasible and active (207). However, existing differences among countries have been reported (208). In consideration of the multiple and challenging clinical variables (e.g. comorbidities, high-grade tumors, advanced stage), an optimal care of elderly patients with a diagnosis of SGC should start with a multidisciplinary tumor board evaluation, together with a G8 questionnaire, and a comprehensive geriatric assessment should be offered whenever needed (**Figure 2**).

Surgery is the hinge of curative pathway in SGC patients, and not less so in the elderly population. An advanced chronological age is not an absolute contraindication for surgery. The tumor resectability, the extent of surgery and the expected outcomes should be discussed. In addition, screening tools such as the Age Adjusted Charlson Comorbidity Index and G8 questionnaire have to be assessed also in this setting, in order to identify frail patients and their special needs.

Postoperative RT is generally delivered in advanced stage, high-grade or in case of high-risk pathological features. The choice of radiation type – by photons or heavy particles – should be based on clinicopathological characteristics, resection margins, and facilities availability. Toxicities should be closely monitored and early managed, with a careful and continuous supportive care during the whole treatment period. For those patients not amenable to surgery, either due to the extent of disease or comorbidities, exclusive RT with radical or palliative aim would be most reasonable choice. Based on the available data, there is no evidence to support that elderly patients undergoing particle therapy should be treated differently than younger subjects. In fact, toxicity profiles and control rates favor particle therapy over photon RT. There is a general lack of information on structured quality of life assessments in the particle therapy literature throughout all age groups, and this subject should be addressed alongside future prospective clinical trials. Until further data are available, no recommendations can be made on differential treatments with particle therapy in elderly patients with SGCs.

The use of systemic chemotherapy, especially cisplatin-based combinations, might face some barriers in elderly patients. The choice of systemic therapy should be independent from the chronological age, but based on performance status, comorbidities and also caregivers’ support. In the last years, as a comprehensive molecular characterization of SGCs has been developed, the therapeutic options have potentially expanded, following the principles of precision oncology. However, differently from the patients affected by high-incidence malignancies harboring analogous molecular alterations, patients with molecularly targetable SGCs are penalized by the scarcity of clinical trials specifically dedicated to these rare cancers, and they can access to innovative cancer drugs mostly through basket clinical trials, managed access programs or off-label. The type of primary tumor has to be considered in the algorithm choice; for instance, taxane and gemcitabine are not recommended in AdCC due to the lack of activity. Personalized approaches are feasible, especially for some histotypes as SDC, adenocarcinoma NOS and ca ex-PA. The search for known molecular alterations (e.g. AR and HER2 in SDC and ca ex-PA) is recommended to offer an effective therapeutic alternative to chemotherapy (e.g. ADT in case of AR overexpression; HER2-blockade in case of HER2 overexpression; NTRK inhibitors in case of NTRK fusions).

**Figure 3:**
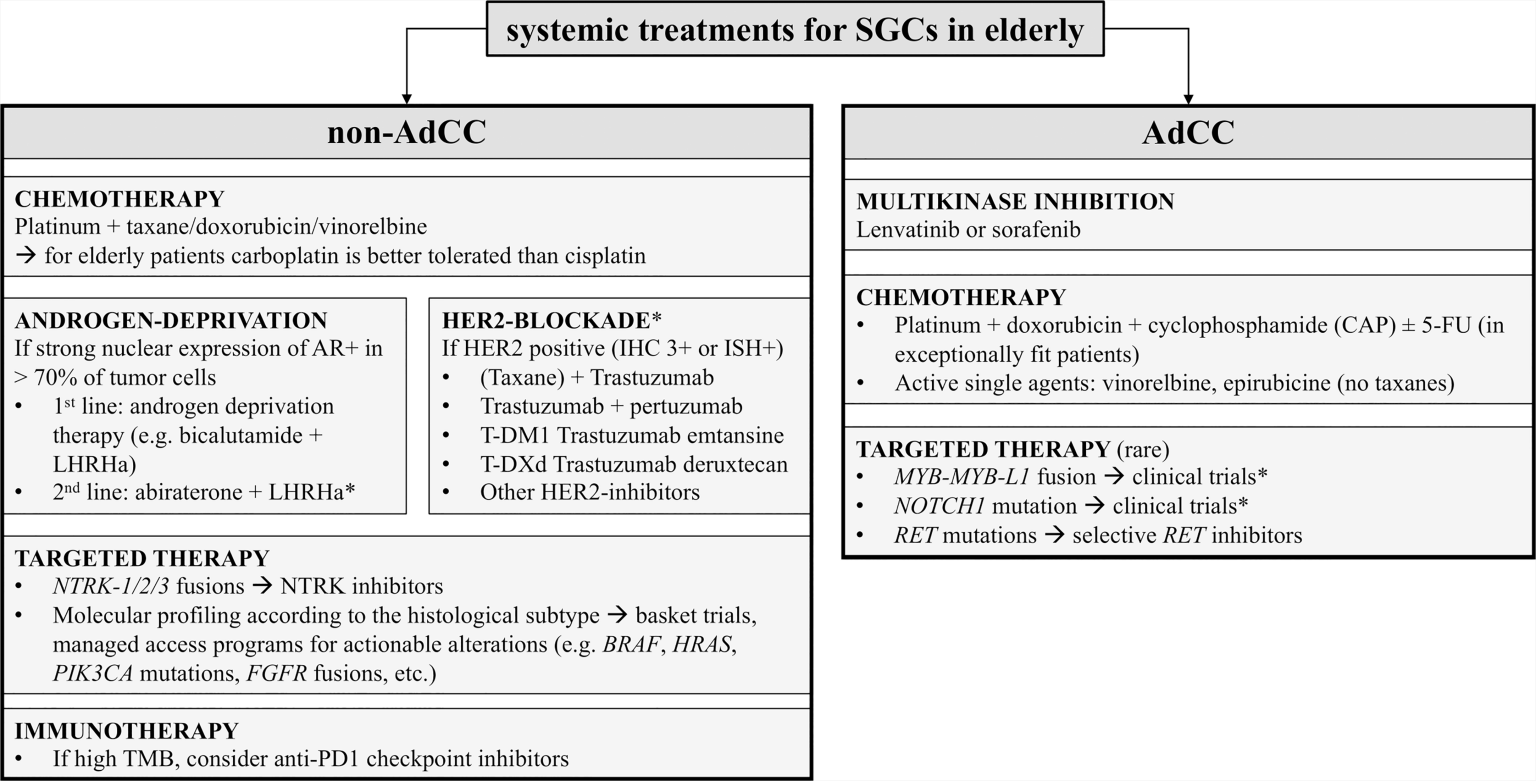
The main strategies for the treatment of patients with salivary gland cancers (AdCC and non-AdCC histotypes) readapted to the elderly population. Therapies marked with (*) have shown activity in clinical trials but are not currently licensed for R/M SGC indication. AR, androgen receptors; LHRH, luteinizing hormone-releasing hormone analogue; HER2, human epidermal growth factor receptor 2, T-DM1, trastuzumab emtansine; NTRK, neurotrophic tyrosine receptor kinase.

Next Generation Sequencing is the most cost-effective approach to find further actionable targets, and this technology can be available in referral cancer centers and/or by prescreening of innovative targeted therapy-oriented clinical trials. The availability of new drugs may change among countries, and the participation of fit elderly patients into clinical trials is encouraged (**Figure 3**). Overall, the management of elderly patients with SGCs is extremely challenging and requires a complex multidisciplinary know-how. The referral to experienced facilities is recommended also for elderly patients, in order to guarantee the most accurate diagnosis and therapeutic opportunities.

## Author contributions

EC, CVL, AZ, AJ, GG, FD, LFL, VG, VVP and LDL participated in the concept design of the manuscript. EC and LDL drafted the introduction and discussion sections, developed further by all authors. Each subsection on treatments was written by specialists of the fields: Epidemiology by GG and FD. Surgery by CVL and VVP. Radiation therapy by VG and AZ. Particle therapy by AJ. Systemic therapy by EC, LFL and LDL. All authors contributed to the revision of the final manuscript and approved the submitted version.

## Funding

VVP and CVL disclosed a financial support for the publication of this article from the Walter Vandeputte Head and Neck Cancer Fund (KU Leuven, Leuven, Belgium). The remaining authors received no financial support for the publication of this article.

## Conflict of interest

Author LFL received occasional conference honoraria/Advisory Board fees from Astrazeneca, Bayer, MSD, Merck-Serono, AccMed, Neutron Therapeutics, Inc. Author LDL received conference honoraria/Advisory Board fees from EISAI, MSD, Merck Serono, McCann Healthcare, Eli Lilly, Sanofi, Sunpharma, IPSEN, Bayer, Roche. Author VVP performed consultancy services for Bristol-Meyers Squibb 2019 and for ATOS medical 2020 and is a surgical trainer for thyroid surgery for Ethicon – J&J. None of these relationships interfere with the intellectual content in the current manuscript.

The remaining authors declare that the research was conducted in the absence of any commercial or financial relationships that could be construed as a potential conflict of interest.

## Publisher’s note

All claims expressed in this article are solely those of the authors and do not necessarily represent those of their affiliated organizations, or those of the publisher, the editors and the reviewers. Any product that may be evaluated in this article, or claim that may be made by its manufacturer, is not guaranteed or endorsed by the publisher.

## Glossary

**Table:**

ACCI	Age-Adjusted Charlson Comorbidity Index
ACE-27	Adult Comorbidity Evaluation index
AcCC	Acinic cell carcinoma
ADC	Antibody-drug conjugate
AdCC	Adenoid cystic carcinoma
ADT	Androgen deprivation therapy
AE	Adverse event
AFIP	Armed Forces Institute of Pathology
AJCC	American Joint Committee on Cancer
AR	Androgen Receptor
ASCO	American Society of Clinical Oncology
BSC	Best supportive care
Ca ex-PA	Carcinoma ex-pleomorphic adenoma
CAMSG	Cribriform adenocarcinoma of minor salivary glands
CGA	Comprehensive Geriatric Assessment
ChT	Chemotherapy
CNB	Core needle biopsy
CT	Computed tomography
CTCAE	Common terminology criteria for adverse events
DFS	Disease-free survival
DHT	Dihydrotestosterone
EMA	European Medicines Agency
ENE-	Without extra-nodal extension
ENE+	With extra-nodal extension
ESMO	European Society for Medical Oncology
FDA	Food and Drug Administration
FGF	Fibroblast growth factor
FGFR	Fibroblast growth factor receptor
FNAB	Fine needle aspiration biopsy
GAN	Greater auricular nerve
Globocan	Global Cancer Observatory
GnRH	Gonadotropin-releasing hormone
GyRBE	Gray relative biological effectiveness
HER2	Human epidermal growth factor receptor 2
HNC	Head and Neck cancer
HNSCC	Head and Neck Squamous Cell Carcinoma
HR	Hazard ratio
ICD-O	International Classification of Diseases for Oncology
ICI	Immune checkpoint inhibitors
IGF	Insulin-like growth factor
IHC	Immunohistochemistry
IMRT	Intensity-modulated radiation therapy
ISH	In situ hybridization
LET	Linear energy transfer
LHRHa	Luteinizing hormone releasing hormone analogues
LCR	Locoregional control
MAPK	Mitogen-activated protein kinase
MASC	Mammary analogue secretory carcinoma
MEC	Mucoepidermoid carcinoma
MKI	Multikinase inhibitor
MNA	Mini Nutritional Assessment
mPFS	Median progression free survival
MRI	Magnetic resonance imaging
MSG	Major salivary gland
mSG	Minor salivary gland
mSGC	Minor salivary gland cancer
MSI-H	High microsatellite instability
MDT	Multidisciplinary Team
MyoEpi	Myoepithelial carcinoma
NCCN	National Comprehensive Cancer Network
NGS	Next-generation sequencing
NOS	Not otherwise specified
ORR	Objective response rate
OS	Overall survival
PA	Pleomorphic adenoma
PAC	Polymorphous adenocarcinoma
PDGFRα	Plateled-derived growth factor receptor α
PD-L1	Programmed death-ligand 1
PFS	Progression free survival
PLGA	Polymorphous low-grade adenocarcinoma
PORT	Postoperative radiation therapy
QoL	Quality of life
QW	Once weekly
RBE	Relative biological effectiveness
R/M	Recurrent/metastatic
RT	Radiotherapy
SBRT	Stereotactic body radiation therapy
SC	Secretory carcinoma
SCAIF	Supraclavicular artery island flap
SCC	Squamous cell carcinoma
SCGFR	Stem cell growth factor receptor
SDC	Salivary duct carcinoma
SEER	Surveillance Epidemiology and End Results
SGC	Salivary gland cancer/carcinoma
SGT	Salivary gland tumor
SIB	Simultaneous integrated boost
T-DM1	Ado-trastuzumab emtansine
T-Dxd	Trastuzumab deruxtecan
TMB	Tumor mutational burden
TNM	Tumor/Node/Metastasis
TRK	Tropomyosin receptor kinas
UICC	Union for International Cancer Control
VEGF	Vascular endothelial growth factor
VMAT	Volumetric modulated arc therapy
WHO	World Health Organization
